# Ce^3+^-ion, Surface Oxygen Vacancy, and Visible Light-induced Photocatalytic Dye Degradation and Photocapacitive Performance of CeO_2_-Graphene Nanostructures

**DOI:** 10.1038/s41598-017-06139-6

**Published:** 2017-07-19

**Authors:** Mohammad Ehtisham Khan, Mohammad Mansoob Khan, Moo Hwan Cho

**Affiliations:** 1 School of Chemical Engineering, Yeungnam University, Gyeongsan-si, Gyeongbuk 38541 South Korea; 2 Chemical Sciences, Faculty of Science, Universiti Brunei Darussalam, Jalan Tungku Link, Gadong BE1410 Brunei Darussalam

## Abstract

Cerium oxide nanoparticles (CeO_2_ NPs) were fabricated and grown on graphene sheets using a facile, low cost hydrothermal approach and subsequently characterized using different standard characterization techniques. X-ray photoelectron spectroscopy and electron paramagnetic resonance revealed the changes in surface states, composition, changes in Ce^4+^ to Ce^3+^ ratio, and other defects. Transmission electron microscopy (TEM) and high resolution TEM revealed that the fabricated CeO_2_ NPs to be spherical with particle size of ~10–12 nm. Combination of defects in CeO_2_ NPs with optimal amount of two-dimensional graphene sheets had a significant effect on the properties of the resulting hybrid CeO_2_-Graphene nanostructures, such as improved optical, photocatalytic, and photocapacitive performance. The excellent photocatalytic degradation performances were examined by monitoring their ability to degrade Congo red ~94.5% and methylene blue dye ~98% under visible light irradiation. The photoelectrode performance had a maximum photocapacitance of 177.54 Fg^−1^ and exhibited regular capacitive behavior. Therefore, the Ce^3+^-ion, surface-oxygen-vacancies, and defects-induced behavior can be attributed to the suppression of the recombination of photo-generated electron–hole pairs due to the rapid charge transfer between the CeO_2_ NPs and graphene sheets. These findings will have a profound effect on the use of CeO_2_-Graphene nanostructures for future energy and environment-related applications.

## Introduction

Visible light-induced photocatalysis using harmless, sustainable, and inexhaustible solar energy is used widely in water purification to remove toxic organic pollutants, such as harmful organic dyes, herbicides, and pesticides^1^. Since the innovation of water splitting under UV light irradiation by Honda and Fujishima^1, 2^, semiconductor photocatalytic technology using solar energy has been studied widely to understand the purification of polluted water, production of renewable hydrogen energy from water splitting, and the removal of harmful microorganisms^2, 3^. The TiO_2_ semiconductor is one of the most widespread photocatalysts owing to its low cost, non-toxicity, relatively high chemical stability, and strong oxidizing power^4–9^. On the other hand, the wide band gap (3.2 eV) of TiO_2_, which means that it can only utilize ultraviolet light that makes up about 4% of solar energy, and low quantum efficiency limits its use in large scale applied applications^3, 10^. To resolve these drawbacks of TiO_2_, considerable efforts have been made to improve its photocatalytic ability and to extend its light-response range, such as doping^11, 12^, and cocatalyst loading^13^. Nevertheless, there are still many problems to solve and it is urgent and critical to develop an efficient, sustainable, and stable photocatalyst that is driven by visible light^14–17^, which accounts for approximately 45% of solar energy^18^. Over the past few years, considerable efforts have been made towards the development of highly effective and robust photocatalysts for the oxidative degradation of organic dyes to harmless end-products, such as CO_2_, and H_2_O^11, 18^. Nanosized metal oxides (TiO_2_, SnO_2_, CeO_2_, and WO_3_) and their composites with carbon based materials have been reported to have electronic properties with excellent recyclability owing to their extended surface areas with enhanced photocatalytic activities^11, 18–24^. The finding of graphene, which is a flat single layer of carbon atoms with a perfect sp^2^-hybridized two-dimensional structure, has led researchers to investigate composites of graphene with other nanomaterials, such as noble metals, complex oxides, and metal oxides^25^. Among them, graphene-metal oxide nanostructures have attracted particular attention as a viable way to boost the efficiency of various photocatalytic and photocapacitive applications^25–27^.

Ceria (CeO_2_) is a favorable rare earth metal oxide material that has been assessed for many technological applications^28–30^. CeO_2_ NPs are a favorable redox supercapacitor material because it is the most abundant and least expensive rare earth metal oxide with excellent redox characteristics^31, 32^. In addition, the spherical size of nanoparticles with a higher surface area has a prominent effect on the pseudo-capacitance^32^. CeO_2_ is a wide band gap semiconductor (~3.4 eV), which limits its use as an electronic material and as a photocatalyst^28^. The chemical functionalization of CeO_2_ is an excellent method for tuning the band structure and the majority carrier type for electronic and optical applications^29, 30^. In particular, defects, such as oxygen vacancies, in CeO_2_ play a crucial role in its photocatalytic properties because these oxygen vacancies can be formed easily and removed rapidly^31, 32^. The oxygen vacancies in CeO_2_ determine the Ce^4+^ to Ce^3+^ ratio, and an increased level of Ce^3+^ leads to enhanced visible light-induced photocatalytic activity by narrowing the band gap of CeO_2_. Oxygen vacancies in CeO_2_ can attach to graphene sheets, which promotes the formation of stable CeO_2_-Graphene nanostructure^33^. A simple strategy is used to improve the visible light photocatalytic properties of CeO_2_ to combine it with conductive carbon-based materials, which include noble metal particles, carbon nanotubes, and graphene matrix^34^. Graphene, which is a two-dimensional matrix with excellent electrical mobility (200000 cm^2^V^−1^s^−1^), extremely large theoretical specific surface area (~2600 m^2^g^−1^), high thermal conductivity (~5000 Wm^−1^K^−1^), and outstanding mechanical properties (high Young’s modulus), has been applied widely as a remarkable support and electron transport material^35–37^. Graphene sheets can be used/act as an efficient electron acceptor to improve photoinduced charge transfer and hinder the backward reaction by separating the evolution sites of hydrogen and oxygen for improved photocatalytic and photocapacitive performance^19^. On the other side, the photocatalytic and photocapacitive properties of graphene nanostructures with rare earth oxides have barely been investigated.

To the best of the author’s knowledge, there has been no research on both the photocatalytic degradation of organic model dye pollutants and photoelectrode studies using Ce_2_O-Graphene nanostructures at the minimum concentration with high performance. In the present study, a simple and scalable synthesis procedure was developed for the fabrication of CeO_2_–Graphene nanostructures through a facile and efficient hydrothermal method. In this method, the hydrothermal treatment provides uniform anchoring/decoration of CeO_2_ NPs on the surface of graphene sheets, which helps improve the photocatalytic and photocapacitive performance of CeO_2_–Graphene nanostructures using the highly conductive graphene sheets network for effective charge transfer. Therefore, this study examined the optical and structural characteristics as well as the photocatalytic degradation ability of the as-synthesized CeO_2_–Graphene nanostructures using the organic model pollutant dye methylene blue and Congo red. The photocapacitive behavior of CeO_2_–Graphene nanostructures as a photoelectrode was also determined by cyclic voltammetry (CV) and electrochemical impedance spectroscopy (EIS) under visible light irradiation in an aqueous solution at room temperature^38–43^. These investigations can promote the further development of CeO_2_–Graphene-based devices for future energy or environment-related applications.

## Results and Discussion

### XRD analysis of pure-graphene, 1 mM and 3 mM CeO_2_-Graphene nanostructures

**Figure 1:**
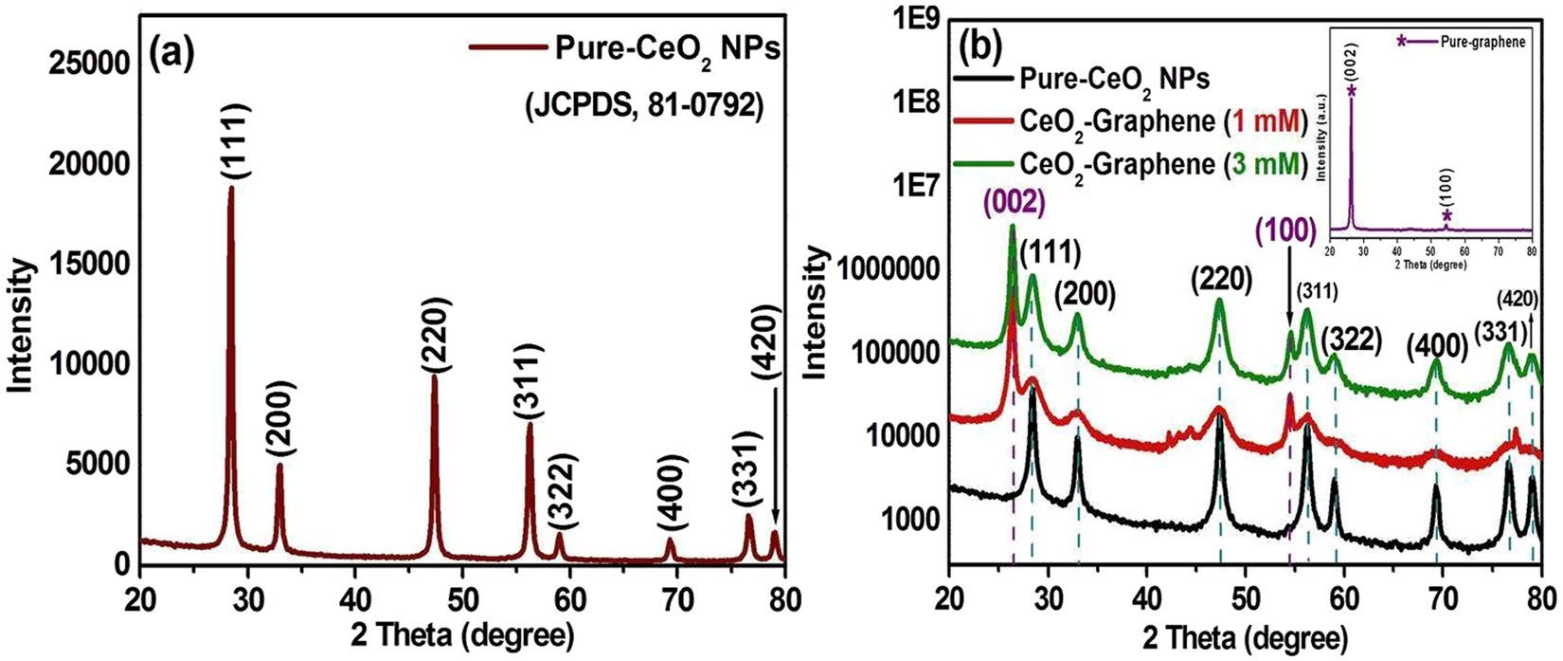
XRD patterns of bare (**a**) CeO_2_ NPs, and, (**b**) CeO_2_-Graphene nanostructure, and inset shows the pure-graphene pattern.

The crystalline phase purity and structural information of the as-synthesized samples were obtained from their XRD patterns. Figure 1(a) shows the XRD pattern of pure-CeO_2_ NPs which was indexed to the fluorite cubic phase of CeO_2_ (JCPDS 34-03494). The XRD peaks of bare CeO_2_ NPs were observed at 28.54°, 33.08°, 47.48°, 56.33°, 59.08°, 69.69° and 79.06° 2θ, which were assigned to (111), (200), (220), (311), (222), (400), (331), and (420) planes respectively^44^. The mean crystallite size of the pure-CeO_2_ NPs was calculated to be 11.8 nm using the Scherer’s formula, D = κλ/β cos θ, where κ is the shape factor that has a typical value of ~0.9, λ is the wavelength (Cu Kα = 0.15405 nm), β is the full width at half maximum of the most intense peak (in radians), and θ is the main peak of pure-CeO_2_ NPs at 28.54° 2θ. The crystallite size of the most intense peak was 11.8 nm (Fig. 1).

The inset in Fig. 1(b) shows the XRD pattern of the graphene matrix, where two foremost peaks can be seen at 26.53° (002) and 54.70° (100). The graphene peaks were well matched and compared with the standard JCPDS No. 01-0646^45^. The XRD pattern of CeO_2_-Graphene nanostructures in Fig. 1(b) revealed the patterns of the as-synthesized CeO_2_ NPs nanostructure demonstrating that CeO_2_ NPs has been anchored successfully to the graphene matrix. Finally, the two prominent peaks, 26.53° (002) and 54.70° (100), were also present in the XRD pattern of the CeO_2_-Graphene nanostructure, which further confirmed the existence and purity of the graphene matrix (Table 1).

**Table 1:** Lattice parameters and crystallite size measured from the XRD patterns of pure-CeO_2_ NPs, 1 mM and 3 mM CeO_2_-Graphene nanostructures.

Samples Name	2θ (degree)	Lattice parameters (Å)	Crystallite size (nm)
Pure-CeO_2_ NPs	28.54	a = b = c = 5.408	2.32
CeO_2_-Graphene nanostructure (1 mM)	28.43	a = b = c = 5.409	2.72
CeO_2_-Graphene nanostructure (3 mM)	28.47	a = b = c = 5.409	2.87

Table 1 lists the experimentally calculated structural parameters, which were obtained from the XRD data. The lattice constant of the as-synthesized 1 millimolar (1 mM) Cerium oxide-Graphene nanostructures and 3 millimolar (3 mM) CeO_2_-Graphene nanostructures samples expanded slightly monotonously. The slight increase in the lattice parameters suggests that lattice defects had formed in the sample due to oxygen vacancies. Extended defects, such as threading dislocations, were also reported to increase the lattice parameters. A small increase in the crystallite size from 2.32 and 2.72 to 2.87 nm indicates the formation of an amorphous phase and defects at the surface of the CeO_2_ crystals. This suggests that the opted synthesis method could be a good method for modifying the required XRD parameters without altering the basic structure (fluorite cubic phase) for the defect-induced band gap narrowing of different metal oxides for the applications, such as the photodegradation of organic dyes and photoelectrodes^44, 46^.

### Raman analysis of pure-graphene, 1 mM and 3 mM CeO_2_-Graphene nanostructures

**Figure 2:**
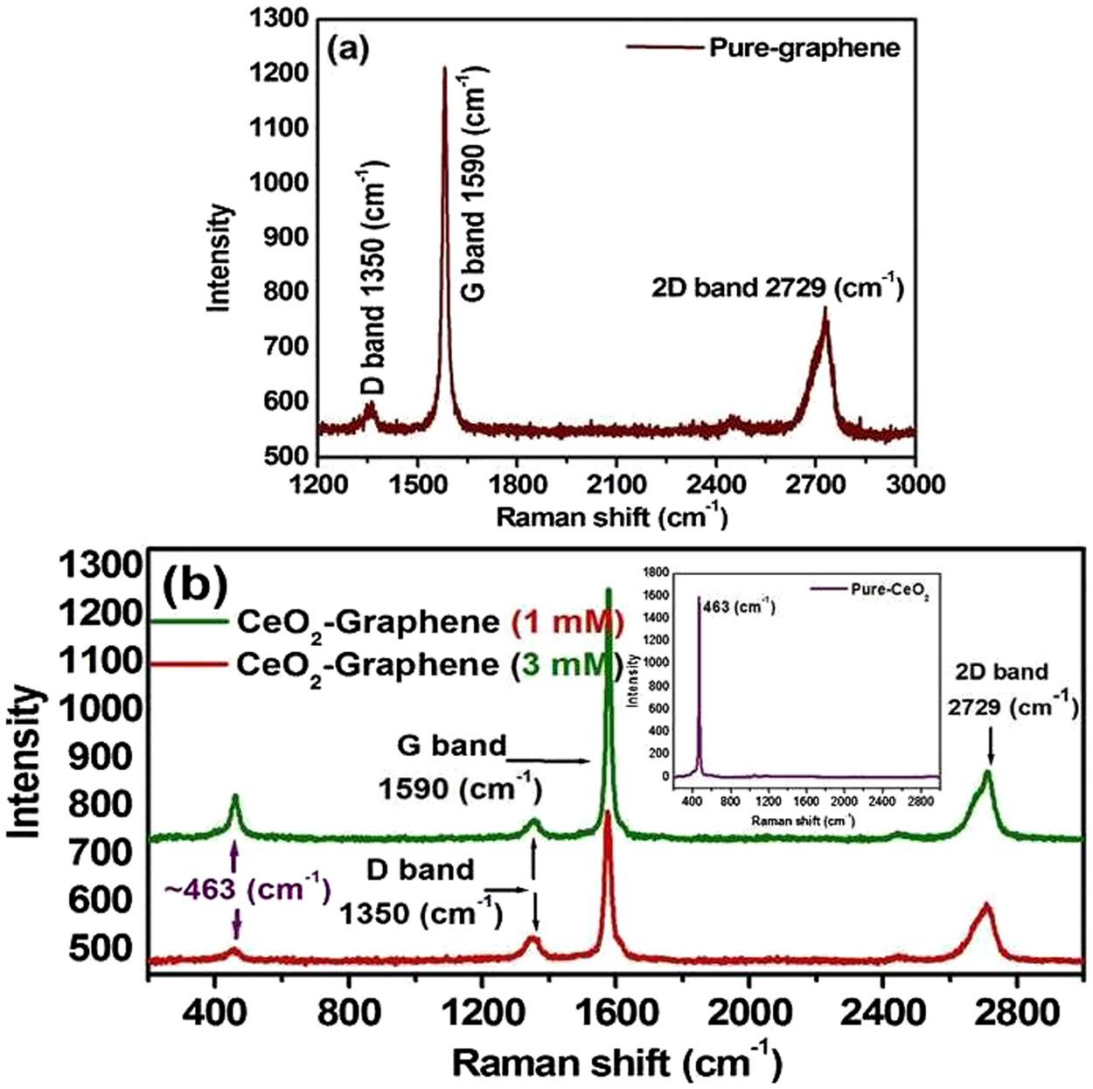
Raman spectrum of (**a**) pure-graphene, and, (**b**) 1 mM and 3 mM CeO_2_-Graphene nanostructures; the inset shows the Raman spectrum of pure-CeO_2_ NPs.

Raman spectroscopy analysis is a non-destructive tool; therefore, it provides valuable information on the electronic and structural properties of graphitic and graphene-related materials, particularly to determine the presence of defects, ordered and/or disordered crystal structures, which are reflected mainly by the vibrational energies of the molecules. Even small changes in the structure can be analyzed by Raman spectroscopy. Figure 2(a) presents the Raman spectrum of the graphene matrix before the CeO_2_ NPs were anchored/decorated to its surface. The Raman spectrum of graphene consisted of three peaks for the D and G bands at 1350 cm^−1^ and 1590 cm^−1^, respectively, and for the 2D band peak at 2729 cm^−1^ ^46^. Similar characteristic peaks of the D, G bands at approximately 1350 cm^−1^ and 1590 cm^−1^ and a broad 2D band 2729 cm^−1^ were observed for the CeO_2_-Graphene nanostructure, as shown in Fig. 2(b) ^45–48^. The intensity of the D (ID) band is related to the vibrational mode of the k-point phonons of A_1g_ symmetry. The G band (ID) corresponds to the tangential stretching mode of the E_2g_ phonon of the sp^2^ carbon atoms, whereas the 2D peak is the secondary D peak that it has the largest intensity in a single/few layer of graphene sheet^49^. In Fig. 2(b), the intensity of the 2D band increased slightly in the case of 1 mM and 3 mM CeO_2_ NPs anchored onto graphene sheets. This further confirms the successful decoration of CeO_2_ NPs onto the graphene sheets (Fig. 2).

The inset of Fig. 2(b), shows the Raman spectrum of pure-CeO_2_ NPs, which exhibits an intense peak at ~463 cm^−1^, corresponding to the symmetrical stretching vibrational mode of the Ce–O_8_ vibrational unit and is assigned to F_2g_^45^. The F_2g_ vibrational mode is quite sensitive to any disorder in the oxygen sublattice, which may result from doping, grain size and thermally induced nonstoichiometric effects. The observed results were matched and comparable to earlier reported studies^50^.

### Optical analysis and band gap calculation of pure-graphene, 1 mM and 3 mM CeO_2_-Graphene nanostructures

**Figure 3:**
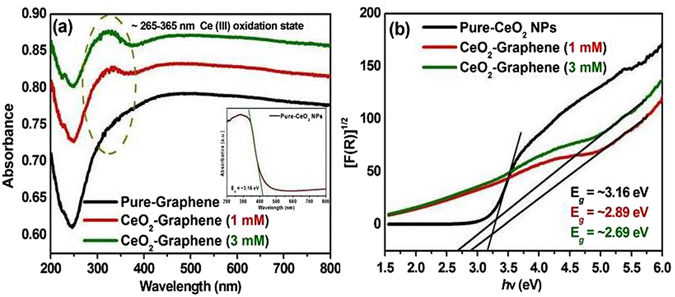
UV–vis, DRS absorption spectra of (**a**) pure-graphene and CeO_2_-Graphene nanostructures. The inset shows the absorbance spectra and band gap of pure-CeO_2_ NPs, and (**b**) optical band gap of 1 mM and 3 mM CeO_2_-Graphene nanostructures.

Optical absorption analysis of the pure-graphene and as-synthesized CeO_2_-Graphene nanostructures were carried out using UV-Vis diffuse reflectance spectroscopy (DRS) at room temperature over the wavelength range, 200–800 nm. In Fig. 3(a), the pure-graphene absorption spectra showed the optical absorption peak without a hump in the 250–370 nm range^45, 46^. After the anchoring/decoration of CeO_2_ NPs onto the graphene matrix, the optical absorption of the 1 mM and 3 mM CeO_2_-Graphene nanostructures increased with a hump (~265–365 nm) and the absorption edge was red shifted considerably compared to that of pure-graphene (Fig. 3).

The dotted line in Fig. 3(a) corresponds to a red shift in the absorption of the as-synthesized nanostructures. This absorption peak (340 to 314 nm) shows the increase in the concentration of Ce ions, suggesting that the particle size is smaller for short time growth^44–48^. The intensity of the absorption peak shifted towards a lower wavelength on decreasing the particle size, meaning that particles with a smaller size exhibit superior visible light absorption behavior. The CeO_2_ NPs show absorption edges at ~400 nm, which are commonly perceived for the Ce^4+^ oxidation state (inset in Fig. 3(a)). In addition, a slight variation and enhancement in absorption band in the UV-Vis spectra of the 1 mM and 3 mM CeO_2_-Graphene nanostructure compared to that of the pure-CeO_2_ NPs was observed. The red-shift in the band gap was attributed to the presence of Ce^3+^ at the grain boundaries, and the band gap decreased with increasing Ce^3+^ concentration, which forms some localized band gap states in the band gap^51^. The absorption band for the 1 mM and 3 mM CeO_2_-Graphene nanostructure between ~265–365 nm was commonly observed for the Ce^3+^ oxidation state. This absorption range further confirmed the successful formation of CeO_2_ NPs in the presence of defects or oxygen vacancies^46, 51^. In addition, a suitable band gap is very important for the photodegradation of organic dyes, photoelectrodes, and photocapacitive applications under visible light. The optical band gap was calculated using the Kubleka-Munk function, as shown in Fig. 3(b) ^46^, as follows:

1 $$F\,({{\rm{R}}}_{\infty })=\frac{{(1-{{\rm{R}}}_{\infty })}^{2}}{2{{\rm{R}}}_{\infty }}=\frac{{\rm{K}}({\rm{\lambda }})}{{\rm{s}}({\rm{\lambda }})}\propto \alpha =\,\frac{{(h{\rm{\nu }}-{{\rm{E}}}_{{\rm{g}}})}^{2}}{h{\rm{\nu }}}$$

where F(R_∞_) is the K-M function or re-emission function; R_∞_ is the diffuse reflectance of an infinitely thick sample; K(λ) is the absorption coefficient; s(λ) is the scattering coefficient; hν is the photon energy. The optical band gap (E_g_) was determined by extrapolating the linear portion (denoted by extended line in Fig. 3(b)) of the plot obtained between [(F(R_∞_)hν)^1/2^] versus hν. The calculated band gap of pure-CeO_2_ and as-synthesized 1 mM and 3 mM CeO_2_-Graphene nanostructure were 3.16, 2.89, and 2.69 eV, respectively. This further shows that the changes in the %R (ESI Fig. S3) and the band gap of the 1 mM and 3 mM of CeO_2_-Graphene nanostructures. The red shift in the band gap is responsible for the formation of some localized band gap states caused by oxygen vacancies and Ce^3+^. The band gap narrowing of the 1 mM and 3 mM of CeO_2_-Graphene nanostructures might be due to oxygen vacancies, defects, and Ce^3+^ increases^44^.

### Photoluminescence studies

**Figure 4:**
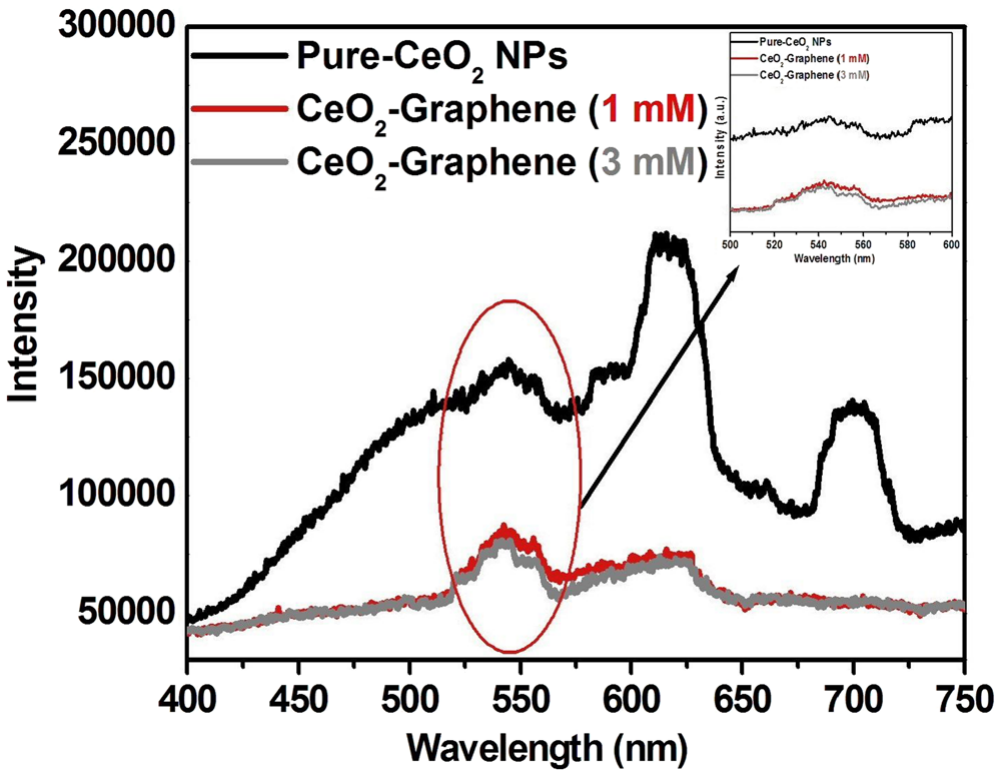
PL spectra of pure-CeO_2_ NPs, 1 mM and 3 mM CeO_2_-Graphene nanostructures.

To understand the outcome of the photogenerated electron-hole pairs at room temperature, photoluminescence (PL) of pure-CeO_2_ NPs, 1 mM and 3 mM CeO_2_-Graphene nanostructures were performed in the range of 400 to 750 nm, as shown in Fig. 4, with an excitation wavelength of 325 nm (Fig. 4).

**Figure 5:**
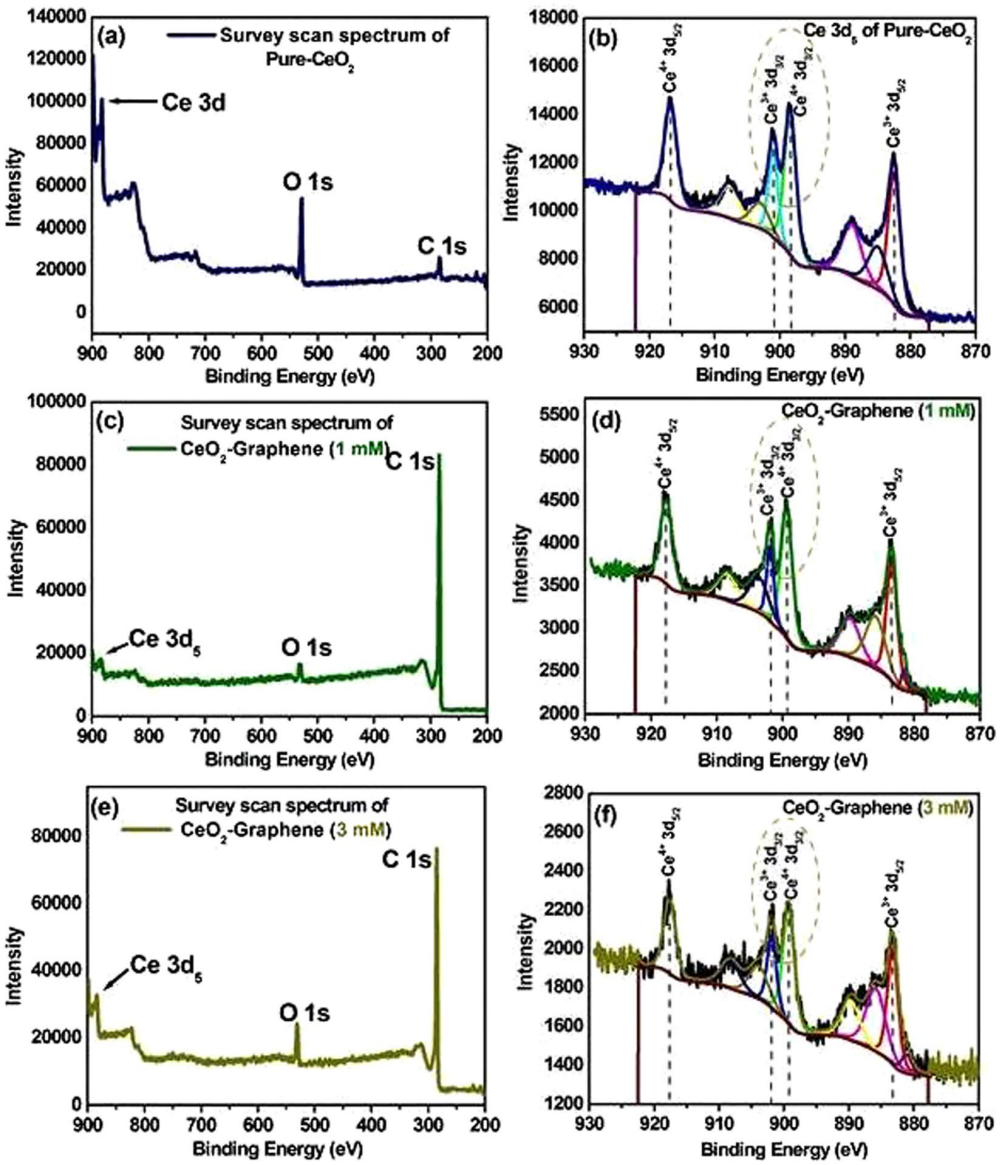
(**a**,**c** and **e**) Survey scan spectra and deconvoluted XPS region spectra (**b**,**d** and **f**) of Ce 3d fitted spectra of pure-CeO_2_ NPs, 1 mM, and 3 mM CeO_2_-Graphene nanostructures.

In general, a higher PL intensity indicates a higher recombination rate of photoexcited hot electrons and holes, which has a negative effect on the photocatalytic degradation performance. In contrast, a lower PL intensity suggests a lower recombination rate, indicating that more photoexcited holes and electrons can participate in the redox reactions, thereby improving the photocatalytic performance of the as-synthesized material^5^. The photoluminescence was observed at 570 to 650 nm after the irradiation of pure-CeO_2_ NPs with a He-Cd laser (λ_ex_ = 325 nm). In this case, however, under the experimental conditions for the as-synthesized CeO_2_-Graphene nanostructures, a lower PL intensity graph recorded indicates a lower recombination rate of electrons. This is due to the presence of surface defects or oxygen vacancies in the CeO_2_ NPs^44, 45, 48^. At higher concentrations of Ce^3+^ states and a corresponding high concentration of oxygen vacancy states, a higher percentage of valence electrons can be excited to the defect state or oxygen vacancy states, which would lead to a lower PL signal intensity as observed in the case of the 3 mM CeO_2_-Graphene nanostructure. The presence of these oxygen vacancies was also confirmed by XPS (Fig. 5(b and c)). Oxygen vacancies can serve as an electron trap, which will reduce the recombination of photogenerated electrons and holes, thereby promoting photocatalytic degradation performance^45, 46^.

### X-ray photoelectron spectroscopic analysis of pure-graphene, pure CeO_2_ NPs and 1 mM and 3 mM CeO_2_-Graphene nanostructures

To confirm the changes in surface valence chemistry and oxidation state in nanoceria, X-ray photoelectron spectroscopy (XPS) was carried out for the pure-CeO_2_ NPs and 1 mM and 3 mM CeO_2_-Graphene nanostructures in the region of 200–900 eV (Fig. 5).

Figure 5(a) shows the XPS survey scan spectrum of the pure-CeO_2_ NPs, in which two major peaks were assigned to Ce and O and a small peak of carbon (C 1s), which might be due to instrumental error. In addition, no contaminant peak was detected within the sensitivity of the technique. The binding energy (BE) of Ce 3d_5_ and O 1 s was recorded at 882.8 and 530.2, respectively^52^. Figure 5(b) shows the fitted spectrum of pure-CeO_2_ NPs, in which four major peaks can be seen clearly. The Cd 3d peaks were confirmed by XPS analysis: Ce^4+^ 3d_5/2_, Ce^3+^ 3d_3/2_, Ce^4+^ 3d_3/2_ and_._ Ce^3+^ 3d_5/2_. The main characteristic peaks of Ce^4+^ 3d_5/2_ and Ce^4+^ 3d_3/2_ were observed at 916.7 and 898.4 eV, respectively, whereas the peaks located at 901.1 and 882.6 eV were assigned to Ce^3+^ 3d_3/2_ and Ce^3+^ 3d_5/2_, respectively. These spectral data are fully consistent with previous reports^53^. Figure 5(c) shows the survey scan spectra of the as-synthesized 1 mM CeO_2_-Graphene nanostructure, which displays a small peak for Ce 3d_5_ and O 1 s at a binding energy of 882.8, and 530.2 eV, which confirms the successful formation and anchoring of the CeO_2_ NPs to the graphene matrix. The sample yielded a high intensity peak of C1s at 284.6 eV, which was assigned to sp^2^-bonded carbon (C-C), indicating the successful formation of 1 mM of CeO_2_-Graphene nanostructure^19^ (Fig. 5(d)). The XP spectrum consists of four distinct signals of Ce 3d_5_, corresponding to different ionic states of Ce^4+^ and Ce^3+^. The characteristic XPS signals of Ce^4+^ 3d_5/2_, and Ce^3+^ 3d_5/2_ electronic states were observed at 916.7 and 882.6.4 eV, respectively. The two main peaks at 901.1 and 899.2 eV were assigned to Ce^3+^ 3d_3/2_ and Ce^4+^ 3d_3/2_, which represent the relative amount of the Ce^3+^ and Ce^4+^ electronic state concentrations present in the sample. From the intensity of the respective peaks, the concentration of Ce^3+^ was comparatively higher in the sample analyzed compared to the peak intensity of pure-CeO_2_ NPs. This confirms the presence of oxygen vacancy/defects in CeO_2_ in the CeO_2_-Graphene nanostructure (1 mM)^54^. Figure S4(a) shows the fitted peak of C 1s at 284.6 eV, which corresponded to the value reported in the literature^46^. Figure 5(e) shows similar photoelectron peaks indicated for Ce 3d, O 1 s, and C 1s at binding energies of 882.8, 530.2 eV, and 284.6 eV, respectively. Minor enhancement in the peak intensity of Ce 3d and O 1 s, which is due to the increased concentration of the CeO_2_ precursor. The C 1s peak confirmed the presence of sp^2^ carbon atoms related to the graphene matrix. Figure 5(f) similarly shows the main peaks of Ce^4+^ 3d_5/2,_ Ce^3+^ 3d_3/2,_ Ce^4+^ 3d_3/2_ and Ce^3+^ 3d_5/2_ at binding energies of 916.7, 901.1, 899.2, and 882.6.4 eV, respectively. The main focus is the concentration and intensity of Ce^3+^ 3d_3/2_ and Ce^4+^ 3d_3/2_. As shown from the Ce^3+^ 3d_3/2_ peak, the intensity of Ce^3+^ increased with increasing concentration (3 mM compared to the 1 mM CeO_2_-Graphene nanostructure). As indicated by XPS, the 1 mM and 3 mM CeO_2_-Graphene nanostructures contained a large number of defects and Ce^3+^ oxidation states^44, 54^, which might lead to the formation of a surface state energy band of oxygen. Figure S4(b) exhibits the fitted peak of C 1s at 284.6 eV, which was well coordinated with the as-described value in the literature^46^. The oxygen adsorption, desorption, and diffusion processes might occur easily on the surface of the 1 mM and 3 mM of CeO_2_-Graphene nanostructures, which can greatly improve their optical properties and impart visible light-induced photocatalytic degradation of organic dyes and photoelectrodes response^44^ (Table 2).

**Table 2:** Atomic weight percent of the elements with binding energy values.

No.	Elements	Binding energy (eV) (1 mM and 3 mM)		Atomic weight (%) (1 mM and 3 mM)		Fitted peak percentage of Ce^4+^ and Ce^3+^	
1	Carbon	284.6	284.6	93.37	88.47	88.37	84.45
						11.63	15.55
2	Oxygen	530.2	530.2	5.71	9.24	59.04	33.72
						40.96	66.28
3	Cerium Ce 3d_5_	882.8	882.8	0.93	2.29	18.21 (Ce^4+^)	21.91 (Ce^4+^)
						13.32 (Ce^3+^)	18.10 (Ce^3+^)

Table 2 lists the elemental confirmation and atomic weight% of the elements, such as carbon, oxygen and cerium, which were obtained from XPS analysis. The higher atomic weight percent of carbon clearly shows the presence of graphene sheets on which spherical-shaped CeO_2_ NPs are decorated^54^. This was attributed to the combined effect of CeO_2_ NPs and graphene, which imparted improved performance to the CeO_2_-Graphene nanostructure for photocapacitive and photocatalytic activities. In addition, the enhanced peak percentage values of Ce^3+^ further proved the oxygen vacancies and defects in CeO_2_ in the as-synthesized 1 mM and 3 mM CeO_2_-Graphene nanostructures.

### Electron paramagnetic resonance analysis of pure-CeO_2_ NPs, 1 mM and 3 mM CeO_2_-Graphene nanostructures

**Figure 6:**
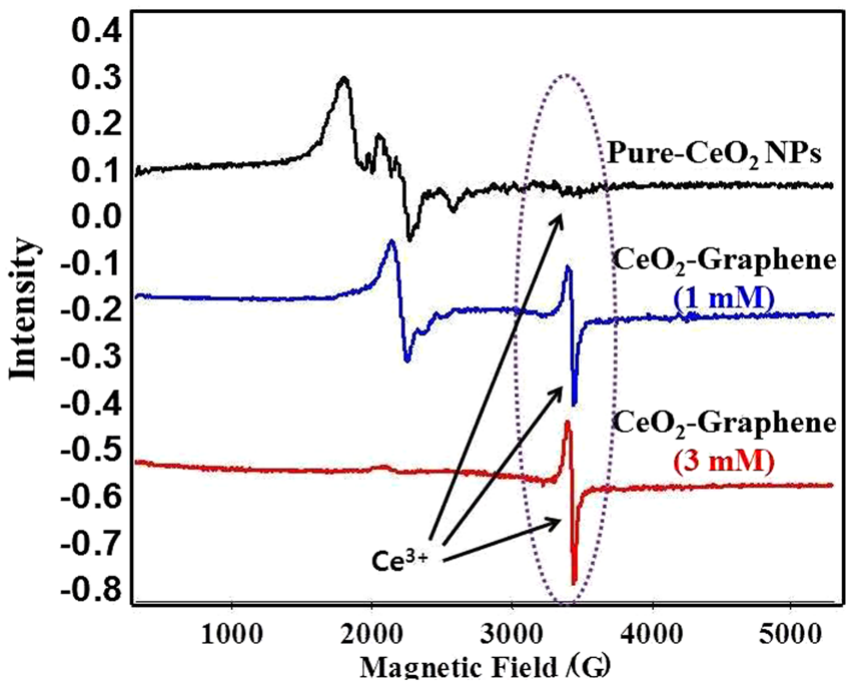
EPR spectra of pure-CeO_2_ NPs and 1 mM and 3 mM CeO_2_-Graphene nanostructures at room temperature with a 100 kHz modulation frequency.

Electron paramagnetic resonance (EPR) spectroscopy is well suited to examining the nature of the oxidation state (Ce^4+^/Ce^3+^) of pure-CeO_2_ NPs and 1 mm and 3 mM CeO_2_-Graphene nanostructures as the powder samples exhibit weak room temperature (RT) ferromagnetism (Fig. 6).

**Figure 7:**
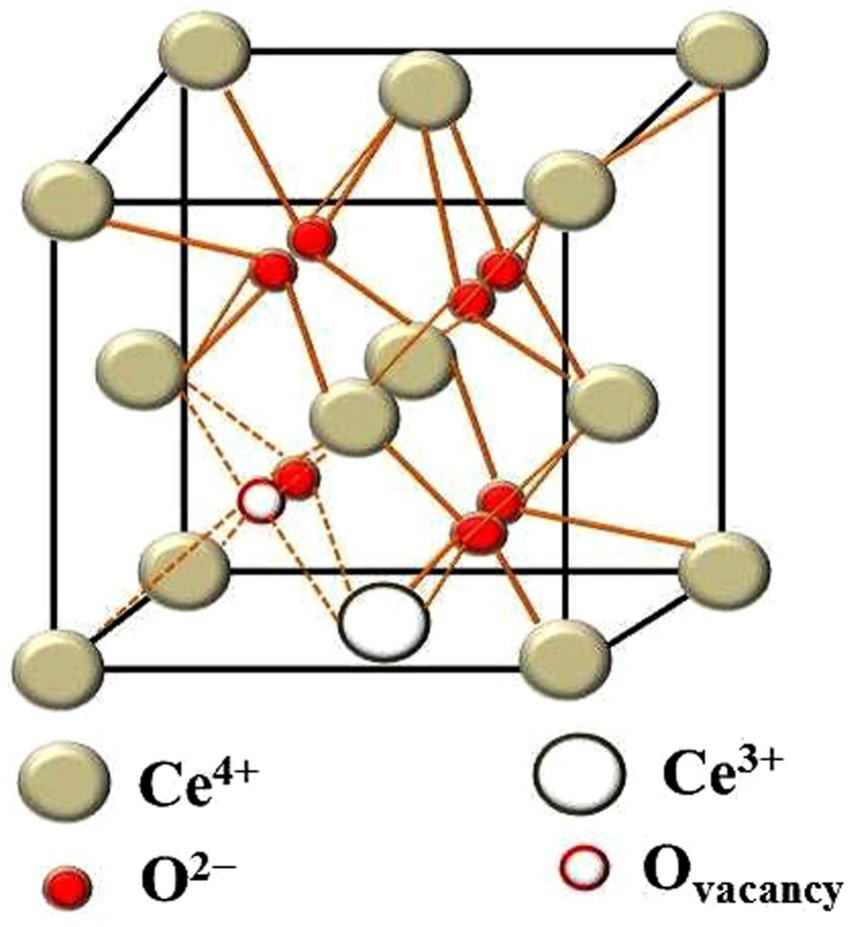
Schematic model of the CeO_2_ structure showing Ce^3+^-ions and oxygen vacancy. The whitish color ball is Ce^4+^; the red color ball is O^2−^ on the lattice site; the red color circled ball is oxygen vacancy; the unfilled white ball represents Ce^3+^ on the lattice site formed after removing oxygen either from surface or from the interior of CeO_2_.

The EPR spectra of pure-CeO_2_ NPs, 1 mM and 3 mM CeO_2_-Graphene nanostructures were recorded (Fig. 6) at RT with a 100 kHz modulation frequency. The pure-CeO_2_ NPs at RT did not show any EPR signals at RT, whereas the EPR signals for the 1 mM and 3 mM CeO_2_-Graphene nanostructure were clearly observed at RT. The EPR signal of the CeO_2_-Graphene nanostructure of 3 mM was much stronger than that of 1 mM at RT^48^. The intensity of the Ce^3+^ signal increases due to the reduction of Ce^4+^ ions to Ce^3+^ following the combination of CeO_2_ with graphene sheets as a support. The intensity of the EPR peaks for Ce^3+^ ions in CeO_2_ was discussed in previous studies and relates mainly to the line with g = ~1.96, which was observed at RT. In addition, g = ~1.96 is close to the g values of the cubic site position of Ce^3+^ in CeO_2_ NPs. The trigonal site of the Ce^3+^-ion can be realized easily near the oxygen vacancy and defects. The 1 mM and 3 mM CeO_2_-Graphene nanostructures showed a single EPR signal at RT, which corresponds to oxygen vacancies, and was very small in the case of pure-CeO_2_ NPs_._ Therefore, EPR showed that the 1 mM and 3 mM CeO_2_-Graphene nanostructures have characteristic paramagnetic properties and oxygen vacancies, which could impart visible light-induced photocatalytic activity^48, 52–54^. Figure 7 presents the schematic observations of the fluorite structure of CeO_2_ along with oxygen vacancies and Ce^3+^ at the lattice sites (Fig. 7).

### Morphological analysis of 1 mM and 3 mM CeO_2_-Graphene nanostructures using transmission electron microscopy

**Figure 8:**
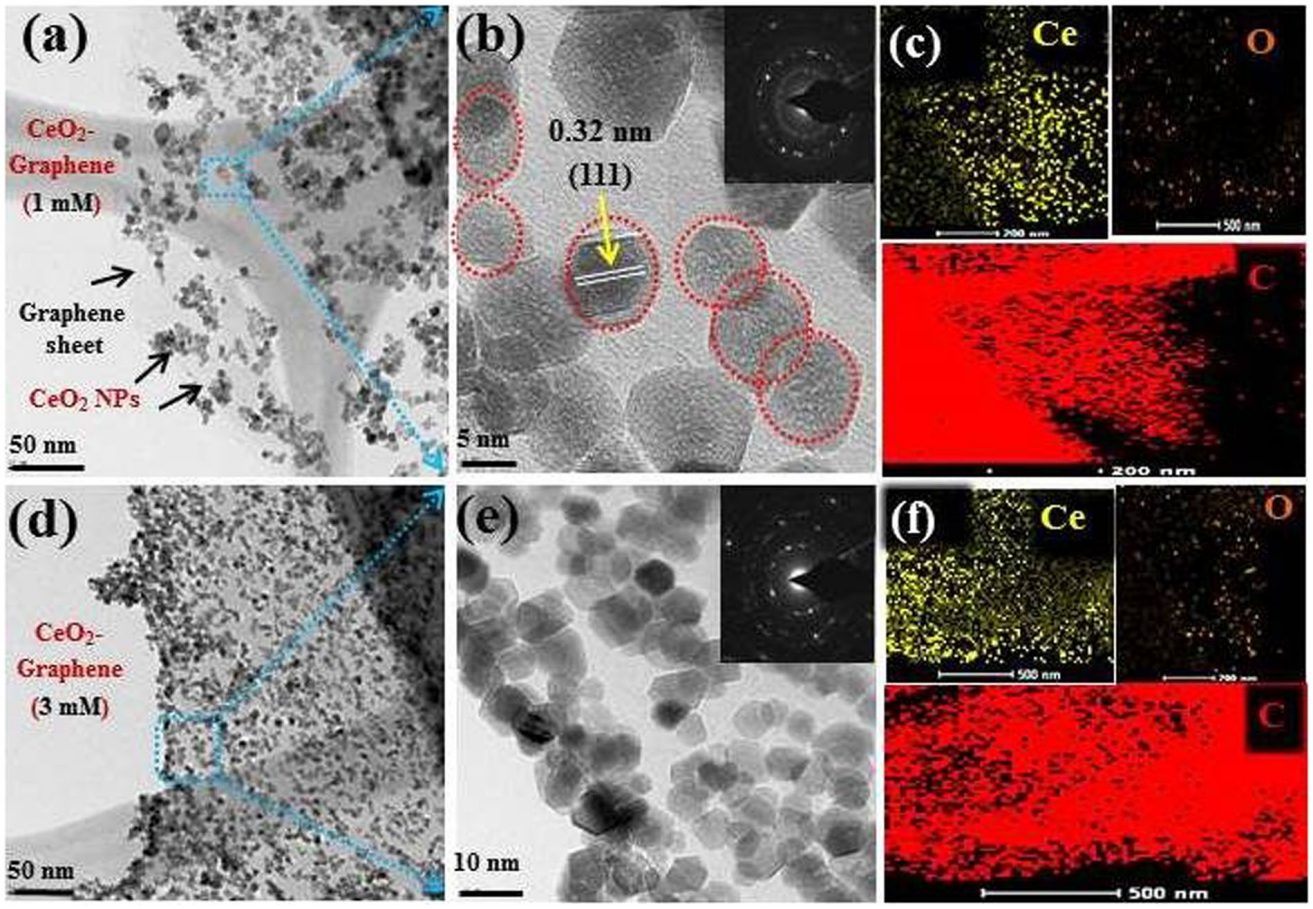
(**a** and **d**) TEM images of the 1 mM and 3 mM CeO_2_-Graphene nanostructures shows the presence of CeO_2_ NPs onto the graphene matrix, (**b** and **e**) HR-TEM image showing the uniform distribution and lattice fringes of CeO_2_ NPs onto the graphene matrix, inset showing the SAED crystal ring pattern (**c** and **f**), showing elemental mapping of Ce (yellow color), O (orange color), and C from graphene in (red color).

The morphology and particle size confirmation of the as-synthesized 1 mM and 3 mM CeO_2_-Graphene nanostructures were characterized by TEM, as shown in Fig. 8.

The two dimensional (2D) sheet-like surface of the graphene matrix was decorated/anchored by CeO_2_ NPs successfully and showed a uniform distribution. In addition, most of the synthesized CeO_2_ NPs were in the range, 10–15 nm, had an almost spherical shape, and were dispersed over the graphene sheets. They presented a good interfacial interaction between the CeO_2_ NPs and graphene sheets. No CeO_2_ NPs were observed outside the graphene matrix. An opaque and single layer graphene sheet was clearly visible from the background of Fig. 8(a) ^54^. A close observation from the HR-TEM image of the 1 mM CeO_2_-Graphene nanostructure displayed in Fig. 8(b) shows that the CeO_2_ NPs were distributed uniformly and homogeneously over the graphene matrix. The interplaner spacing of 0.32 nm was determined, which is consistent with the (111) plane of the CeO_2_ phase calculated from the XRD pattern. The SAED pattern of the nanostructure exhibited a series of bright rings, suggesting that the sample is polycrystalline (inset of Fig. 8(b). The elemental maps presented in Fig. 8(c) shows Ce (yellow), O (orange), and C (red), which provides strong evidence for the coexistence of the CeO_2_ NPs decorated on the graphene sheets. Figure 8(d) clearly show the decoration/anchoring of CeO_2_ NPs to the graphene sheets, which is densely packed with spherical nanoparticles with a uniform distribution. This further confirms the successful decoration of the graphene sheet with small size of CeO_2_ NPs. Figure 8(e) presents HR-TEM image clearly shows nanoparticles within the 10–15 nm range and uniformly distributed over graphene matrix. In 3 mM CeO_2_-Graphene nanostructure case, the CeO_2_ NPs are in large quantity compared to 1 mM CeO_2_-Graphene nanostructure. This also showed that 3 mM is the optimal CeO_2_ NPs concentration for decoration of the graphene matrix. The SAED ring pattern of CeO_2_-Graphene nanostructure (Inset) displays a sequence of bright rings, which suggests that the sample is polycrystalline in nature. Figure 8(f) shows the elemental map in the Ce (yellow), O (orange), and C (red), which provides a solid indication of the existence of CeO_2_ NPs decorated over the graphene sheets. Figure S5(a and b) shows the elemental composition of the 1 mM and 3 mM CeO_2_-Graphene nanostructure. Figure S6(a,b and c) showing the high magnification images of CeO_2_-graphene nanostructures indicating visible lattice fringes and particle size distribution graph of CeO_2_ NPs for 1 mM and 3 mM CeO_2_-Graphene nanostructures.

### Brunauer-Emmett-Teller, specific surface area analysis of pure-graphene and 1 mM and 3 mM CeO_2_-Graphene nanostructures

In order to observe the changes in the specific surface area of the as-synthesized samples, N_2_-BET (Nitrogen adsorption Brunauer-Emmett-Teller) was used. The BET specific surface area analysis of pure-graphene, 1 mM and 3 mM CeO_2_-Graphene nanostructures were measured to be 35.0134 ± 0.0223, 37.1380 ± 0.0999 and 40.8460 ± 0.1068 m^2^/g, respectively. The single point surface area at P/Po was also recorded for pure-graphene, 1 mM and 3 mM CeO_2_-Graphene nanostructures as shown in Table 3. Interestingly, the BET specific surface area of surface oxygen vacancy assisted CeO_2_ samples was increased as the precursor amount increased. This is because of smaller particles having higher surface areas. In the photocatalytic study, higher surface areas and small particles are favored because more active sites are available for molecules that can provide the surface for dye molecules for oxidation or reduction^4, 55, 56^. These results suggest that the visible light photocatalytic performance of CeO_2_-Graphene nanostructures could be improved greatly due to the high concentration of surface donor defects, such as oxygen vacancies, Ce^3+^ centers and increased surface area (Table 3).

**Table 3:** Specific surface area measured from the BET analysis of the pure-graphene, 1 mM and 3 mM CeO_2_-Graphene nanostructures.

Sample name	Single point surface area at P/Po	BET surface area (m^2^/g)
Pure-Graphene	0.300000000:34.3095	35.0134 ± 0.0223
CeO_2_-Graphene nanost ructure (1 mM)	0.300000000:35.0726	37.1380 ± 0.0999
CeO_2_-Graphene nanostructure (3 mM)	0.300000000:39.9112	40.8460 ± 0.1068

## Applications of the 1 mM and 3 mM CeO_2_-Graphene nanostructures

### Evaluation of the photocatalytic degradation of organic model pollutant dyes (CR and MB) using pure-graphene, 1 mM and 3 mM CeO_2_-Graphene nanostructures

**Figure 9:**
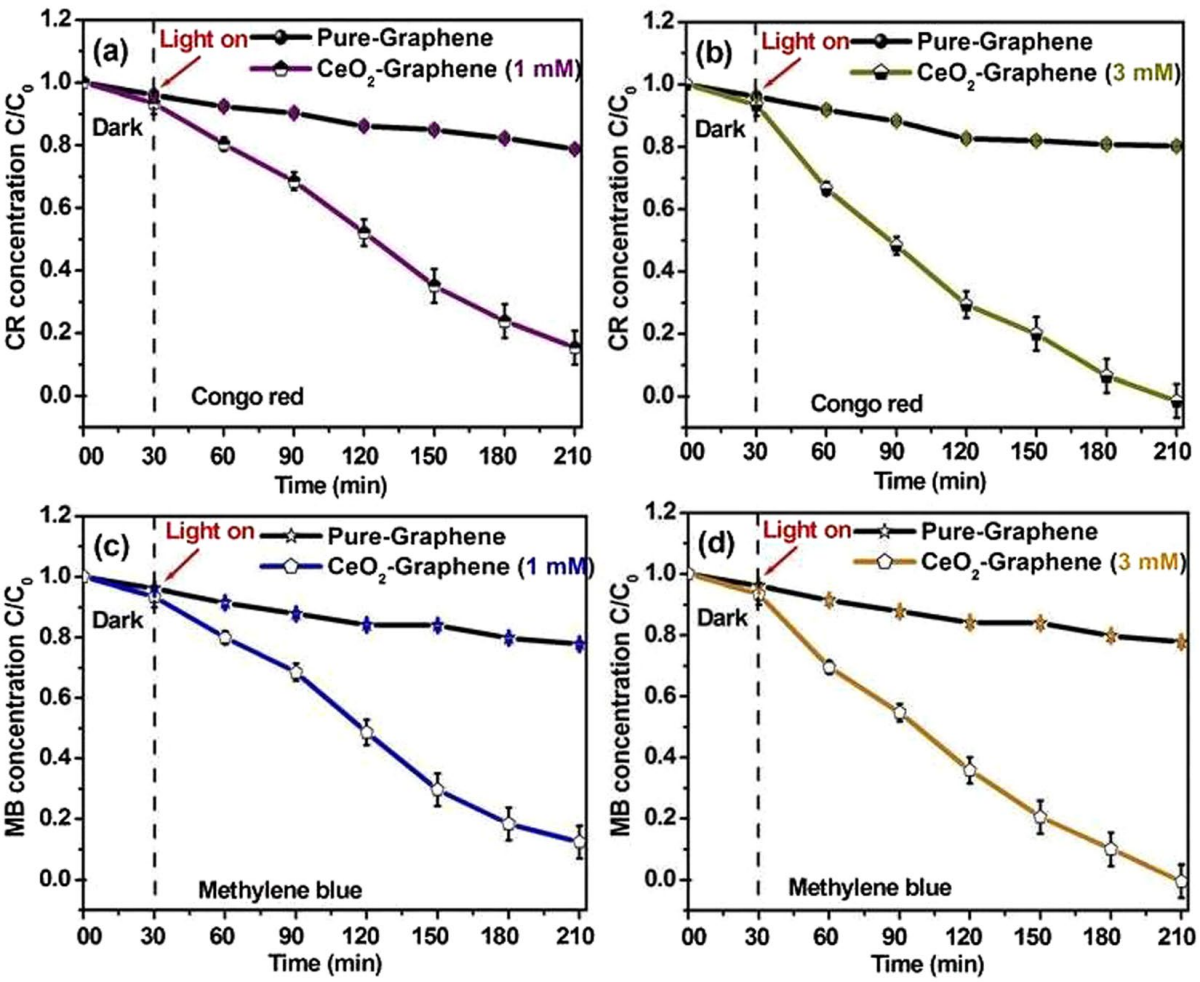
C/C_0_ vs. time (h) plot for the photodegradation/decolorization of (**a** and **b**) CR, and, (b) MB (**c** and **d**) using pure-graphene, and 1 mM and 3 mM CeO_2_-Graphene nanostructures as photocatalyst in the dark and under visible light irradiation.

Metal oxide NPs is a significant reagent used in the pharmaceutical and biochemical industries, whereas different kinds of organic dyes, such as CR and MB, are anionic and cationic hetero polyaromatic model dyes commonly used in the cellulose (textiles) industries (cotton textile, wood pulp, and paper)^46^. The anionic, cationic CR and MB dyes are used widely in a range of fields but their discharge into water can cause environmental pollution. In addition, most of the organic dyes are toxic, carcinogenic, and harmful, resulting in adverse impacts on human and animal health. Towards the better understanding of the significance of this photocatalyst system, the role of a photocatalyst is to accelerate the specific reduction and oxidation (redox) reactions under the visible light illuminated semiconductors^18^. When the semiconductor catalyst was exposed with photons, whose energy is equal to or greater than their band-gap energy (E_g_), an electron (e-cb) is promoted from the valence band (VB) into the conduction band (CB), leaving a hole (h^+^-vb)^19^. Furthermore, the excited electrons and holes transfer to the surface. The rate of recombination is often inhibited by a scavenger or crystalline surface defects, which can easily trap electrons or holes. Therefore, better crystallinity with surface defects can generally decrease the trapping states and recombination sites, subsequent in increased effectiveness in the procedure of the photogenerated carriers for the preferred photoreactions^46^. For higher photocatalytic efficiency, the electron–hole pairs should be separated efficiently, and charges should be transferred rapidly across the surface/interface to restrain the recombination rate^46, 52^. The present photocatalytic degradation of CR and MB were carried out to evaluate the photocatalytic degradation performance of the pure-CeO_2_ and 1 mM and 3 mM CeO_2_-Graphene nanostructures under visible light irradiation using a 400 W lamp (λ > 500 nm). Figure 9(a-d) presents the results of these experiments for CR and MB dye degradation performance. The kinetics of the reaction was plotted as the photodegradation constant of the model dye as ln(C/C_0_) vs. time (t). The photocatalytic degradation rate of anionic CR dye was negligible in the absence of CeO_2_ NPs, which is shown by an analysis of the control (Fig. 9(a and b)). The pure-graphene showed almost negligible degradation performance of ~16%, whereas the photodegradation performances of the 1 mM and 3 mM CeO_2_-Graphene nanostructures as a photocatalyst for the degradation of CR were ~85.2 and ~94.5%, respectively, after 180 min under visible light irradiation, showing almost complete degradation (Fig. 9).

Figure 9(c and d) shows the profile of the photocatalytic degradation ability of the cationic model pollutant dye, MB, using pure-graphene and 1 mM and 3 mM CeO_2_-Graphene nanostructures under visible light irradiation. A small decrease in the rate of degradation of MB was observed in the presence of pure-graphene after visible light irradiation, which suggests that MB is quite stable under visible light^46, 57^. The 1 mM and 3 mM CeO_2_-Graphene nanostructures showed high photodegradation efficiency up to ~85.2 and ~98%, respectively, after 180 min under visible light irradiation. This was attributed to the high surface-to-volume ratio of the small spherical shaped CeO_2_ NPs decorated over the graphene sheets, which helped increase the photocatalytic sites and graphene helps reduce the recombination rate.

Photocatalytic degradation performance of anionic and cationic model pollutant dye CR and MB was recorded higher than that of the reported cationic RhB model pollutant dye with the same nanocomposite^58^. This also proves that graphene is highly efficient for enhancing the catalytic performance of CeO_2_ NPs and behaves as an electron sink, which increases the separation of the photogenerated electron–hole pairs significantly and inhibits their recombination in the presence of the CeO_2_-Graphene nanostructure as a photocatalyst. The variation in the photocatalytic activity of 1 mM and 3 mM CeO_2_-Graphene nanostructures was also supported by DRS (Fig. 3(b)), PL (Fig. 4), XPS (Fig. 5b and c), and EPR (Fig. 6). These results clearly show that the visible light-induced photocatalytic activity of the CeO_2_-Graphene nanostructures has greatly improved because of the narrow band gap and various defects and Ce^3+^ centers.

### Photocapacitive studies of pure-graphene and CeO_2_-Graphene nanostructures

**Figure 10:**
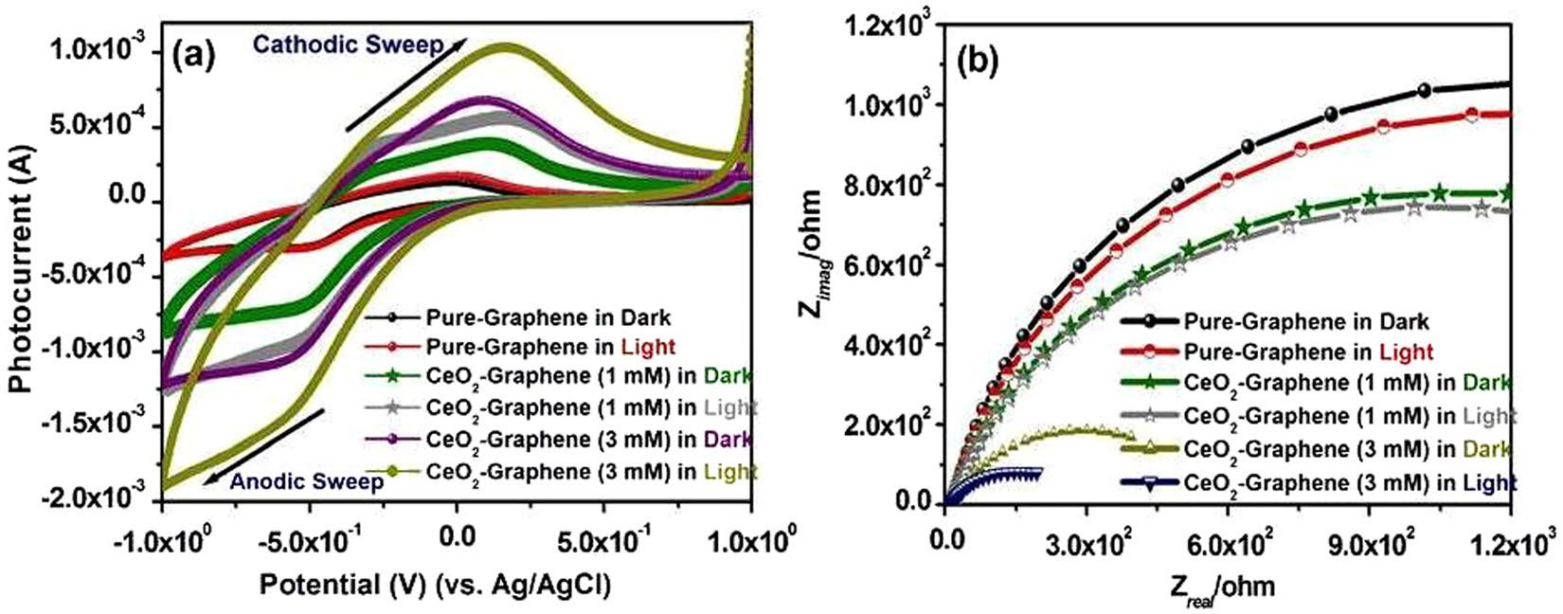
(**a**) Cyclic voltammetry profile, and (**b**) EIS Nyquist plot for the pure-graphene and CeO_2_-Graphene nanostructures in a 0.1 M phosphate buffer electrolyte solution at 25 °C with a scan rate of 0.05 mV/s.

In the ground study of electrochemistry for photoelectrochemical analysis, cyclic voltammetry (CV) is a widely used technique for examining the redox behavior and heterogeneous electron-transfer (HET) rate kinetics as well as defining the electron stoichiometry of any system^19, 59^. CV is generally used to describe the performance of a range of electrical energy storage devices, such as electrochemical capacitors, supercapacitors, photocapacitive device, batteries, and fuel cells^59–61^ (Fig. 10).

Figure 10(a) presents the cyclic voltammograms recorded in the dark and under visible light irradiation at a scan rate of 0.05 mV/s in a 0.1 M phosphate buffer supporting electrolyte on the surface of pure-graphene and 1 mM and 3 mM CeO_2_-Graphene nanostructures photoelectrodes. The photocapacitive performance of the pure-graphene and CeO_2_-Graphene nanostructures were examined systemically and typical CV curves are shown in Fig. 10(a). The CV curves of pure-graphene shows the estimated mirror images with respect to the zero-current line and the rapid current response to the voltage reversal at each end potential (the quasi-rectangular shape and symmetric I–V responses) specifies the ideal pseudocapacitive behavior of the materials. The first anodic peak sweep from 1.0 to −1.0 revealed an oxidation peak area of pure-graphene at 2.128 on the FTO glass surface in the dark and under visible light irradiation. On the other hand, the cathodic sweep from 1.0 to −1.0 revealed a reduction peak area of 2.514 under the same condition. In case of as-synthesized 1 mM and 3 mM CeO_2_-Graphene nanostructures, however, the first cathodic peak was recorded at the oxidation peak area of 8.0844 and 23.668, whereas for the anodic sweep, the peak area was 15.466 and 26.632 in the dark and under visible light irradiation, respectively. Pure-graphene did not show significant enhancement in the oxidation (cathodic sweep) and reduction peaks (anodic sweep) at scan rates of 0.05 mV/s, whereas the as-synthesized 1 mM and 3 mM CeO_2_-Graphene nanostructures showed well-determined oxidation and reduction peaks. The large peak separation (ΔEp) was observed in the case of the CeO_2_-Graphene nanostructures, which was higher than that of pure-graphene. This specifies the improved redox behavior, which could be due to the oxygen vacancy/defects induced CeO_2_ NPs present on the graphene surface. These findings confirm the improved photocapacitive behavior of the CeO_2_-Graphene nanostructures, which is probably due to the small, spherical and uniform decoration of CeO_2_ NPs on the graphene surfaces, which has various defects and Ce^3+^ ions.

### Calculation of the specific capacitance (Csp) behavior of pure-graphene, 1 mM and 3 mM CeO_2_-Graphene nanostructures in the dark and under visible light irradiation

The CV plot (Fig. 10(a)) of the CeO_2_-Graphene nanostructures showed the upgraded positive and negative sweep, specifying their pseudo capacitive behavior. The peak current of the CeO_2_-Graphene nanostructures increased linearly in the dark and under visible light illumination with a positive shift of the cathodic peak and a negative shift of the anodic peak. From the CV graph, the specific capacitances (Csp) of the photoelectrodes were determined using the following equation:

2 $${\rm{Csp}}=2[{\rm{I}}/{\rm{scan}}\,\mathrm{rate}(\mathrm{dv}/\mathrm{dt})\mathrm{VM}]$$

where, I is the average current during the cathodic and anodic sweep, V is the potential and M is the weight of the active material coated on the FTO glass electrode.

From Fig. 10(a), Csp of the pure-graphene was calculated using the above formula in the dark and under visible light irradiation to be 9.25 F g^−1^ and 10.93 F g^−1^ at a current density of 5 mA g^−1^, while the Csp for the 1 mM CeO_2_-Graphene nanostructures was 31.00 F g^−1^ and 59.40 F g^−1^ in the dark and under visible light irradiation. In contrast, for 3 mM, the calculated capacitance was 78.89 F g^−1^ and 177.54 F g^−1^ in the dark and under visible light irradiation. The as-synthesized nanocomposite exhibited enhanced Csp compared to pure-graphene. This was attributed to the highly conductive nature of the graphene sheets, which facilitated the charge transfer of CeO_2_-Graphene nanostructures, and ensured high electrochemical utilization of graphene with Ce^3+^-ions/ defect-induced CeO_2_ NPs^59–61^. Figure 10(b) shows the EIS Nyquist plot (imaginary part, Z′, versus real part, Z″), which is used to examine the supercapacitive performance and typical resistance of the electrode prepared using pure-graphene and 1 mM and 3 mM CeO_2_-Graphene nanostructures. The plots in Fig. 10(b) are composed of a small semicircle at the high frequency region and a line in the lower frequency region. The large semicircle observed for the electrode is indicative of the high interfacial charge-transfer resistance (R_ct_), linking to the deprived electrical conductivity of the materials, whereas the more vertical line is related to an electrode that is closer to an ideal capacitor^62^. On the other hand, as shown in the Fig. 10(b), a significant change in R_ct_ occurs due to the formation of defects in the CeO_2_ NPs as a combination with pure-graphene for the electrode material. In contrast, pure-graphene combined with CeO_2_ NPs shows a smaller R_ct_ because of its good conductivity^57, 62, 63^. This confirms the catalytic effect of CeO_2_, or the synergistic effects of the defect induced CeO_2_ NPs and pure-graphene. In addition, the considerable decrease in the R_ct_ of the electrodes may be related to the improvement in the charge transfer rate at the CeO_2_–Graphene nanostructure.

### Photocatalytic degradation evaluation of the organic model dye using pure-graphene, 1 mM and 3 mM CeO_2_-Graphene nanostructures under visible light irradiation

**Figure 11:**
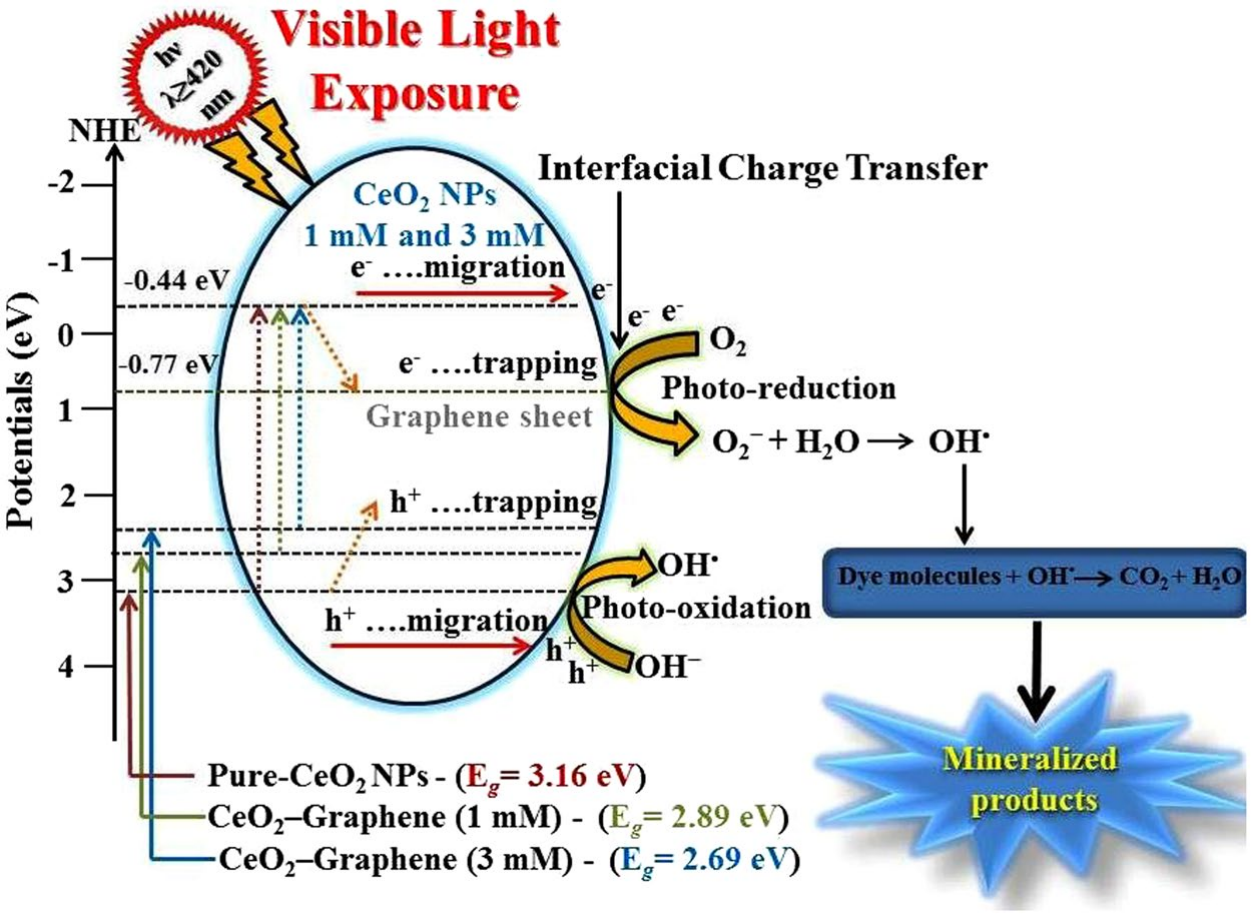
Proposed schematic diagram of the proposed mechanism for the photoexcited electron-hole separation and charge transport process at the CeO_2_-Graphene nanostructures as a photocatalyst under visible light irradiation.

Figure 11 gives a schematic representation of the proposed mechanism for the photocatalytic degradation of CR and MB dye using 1 mM and 3 mM CeO_2_-Graphene nanostructures as a photocatalyst and pure-graphene as a control under visible light irradiation. When the CR and MB dye solution comprising the CeO_2_-Graphene nanostructure is exposed to visible light, electron–hole pairs are generated because of the ejection of an electron from the VB, which makes a hole in the VB. For the duration of the photoexcitation process, some of the ejected electrons transfer to the CB and some electrons are trapped by the localized defect (defects and oxygen vacancies as confirmed by the XPS and EPR results) levels lying below the Ce 4f band^36, 46, 64^. The photogenerated CB electrons are transferred rapidly to the conducting graphene sheets and react with the oxygen dissolved in the dye solution making superoxide radicals (O_2_^**·**^). During this process, graphene sheets act as a trapping center/electron sink and as a co-catalyst. A speedy transfer of the photogenerated electrons from the excited CeO_2_ NPs is the result. Therefore, due to the transfer of electrons from CeO_2_ NPs to the graphene sheets, the recombination rate of photo-generated electron–hole pairs in CeO_2_ NPs is suppressed^46, 64^ (Fig. 11).

XPS and EPR analysis confirms that Ce^3+^-ions and defects are present on the surface of the as-synthesized CeO_2_ NPs. Furthermore, the holes formed in the VB of CeO_2_ are captured by H_2_O or OH^−^ ions and form hydroxyl (OH^**·**^) radicals. These OH^**·**^ radicals interact with the dye molecules and degrade them. After degradation of the dye, these molecules present in the solution can immediately diffuse to the graphene surface *via* π–π conjugation for further degradation. The O_2_^**·**^ and OH^**·**^ radicals can attack the C–S^+^=C functional group of the dye, which is attached to the surface of the catalysts through Coulomb interactions, and degrade the dye to the final products, such as CO_2_, SO_4_^2−^, NO_3_^−^, H_2_O, and H^+^. The complete photodegradation process can be summarized by the following reaction steps:

3 $${{\rm{CeO}}}_{2}+h\nu \to {{\rm{CeO}}}_{2}({{\rm{h}}}^{+}+{{\rm{e}}}^{-})$$

4 $${{\rm{CeO}}}_{2}({{\rm{h}}}^{+})+{{\rm{H}}}_{2}{\rm{O}}\to {{\rm{CeO}}}_{2}+{{\rm{OH}}}^{\cdot }+{{\rm{H}}}^{+}$$

5 $${{\rm{CeO}}}_{2}({{\rm{h}}}^{+})+{{\rm{OH}}}^{-}\to {{\rm{CeO}}}_{2}+{{\rm{OH}}}^{\cdot }$$

6 $${{\rm{CeO}}}_{2}({{\rm{e}}}^{-})+{\rm{Graphene}}\to {{\rm{CeO}}}_{2}+{\mathrm{Graphene}(e}^{-})$$

7 $${\mathrm{Graphene}({\rm{e}}}^{-})+{{\rm{O}}}_{2}\to {\rm{Graphene}}+{{\rm{O}}}_{2}^{\cdot -}$$

8 $${{\rm{OH}}}^{\cdot }+{\rm{Dye}}\,{\rm{molecules}}\,(\mathrm{CR}\,{\rm{and}}\,\mathrm{MB})\to {{\rm{CO}}}_{2}+{{\rm{H}}}_{2}{\rm{O}}+{{\rm{SO}}}_{4}^{2-}\,etc.$$

9 $${{\rm{O}}}_{2}^{\cdot -}+\mathrm{Dye}\,{\rm{molecules}}\,(\mathrm{CR}\,{\rm{and}}\,\mathrm{MB})\to {{\rm{CO}}}_{2}+{{\rm{H}}}_{2}{\rm{O}}+{{\rm{SO}}}_{4}^{2-}\,etc.$$

## Conclusion

CeO_2_ NPs were grown uniformly on graphene sheets using a simple and low-cost hydrothermal method without a surfactant or supporting material. All the synthesized samples were characterized by XRD, Raman, UV-Vis, PL, XPS, EPR, and TEM. TEM and HR-TEM revealed uniform and spherical particles of CeO_2_ NPs, which were decorated uniformly over the graphene sheets. UV-Vis spectroscopy showed that the oxygen vacancy/defects in the CeO_2_-Graphene nanostructures provide a suitable material for the absorption of visible light by decreasing the band gap. XPS and EPR confirmed the defects/oxygen vacancy and paramagnetic behavior of the CeO_2_-Graphene nanostructure. Interestingly, excitation-dependent tunable luminescence behavior could be perceived, which might be reasonably useful for applications in designing new optical devices. Moreover, the photocatalytic performance test of the as-synthesized CeO_2_-Graphene nanostructures showed that the CeO_2_-Graphene nanostructure displayed excellent photocatalytic activity compared to pure-graphene. Within 180 minutes, ~94.5% and ~98% of the CR and MB dye, respectively, had degraded under visible light irradiation. This excellent photocatalytic activity may be due to the synergistic effects of graphene sheets and CeO_2_ NPs (Ce^3+^-ions/defects induced), which are responsible for the smaller optical band-gap values. The photocapacitance investigation indicated that the CeO_2_-Graphene nanostructure, as a photoelectrode, had a maximum photocapacitance of 177.54 F g^−1^ and influenced the regular capacitive behavior. Therefore, the as-synthesized material shows excellent photocatalytic and photocapacitive performance for water treatment and energy storage applications. Based on the improved optical, photocatalytic, and photocapacitive performance, this type of material can be used for real applications, such as real water treatment devices and supercapacitors.

## Experimental Procedures

### Materials used

Cerium nitrate hexahydrate (Ce(NO_3_)_3_ **·**6H_2_O (≥99.99%), cerium oxide (nano CeO_2_) nanostructure, and ammonia (NH_3_) solution (≥99.99%)) were purchased from Sigma Aldrich. Pure-graphene sheets were purchased from Iljin Nano Tech, Seoul, Korea (7–8 layer graphene sheets with a mean length of 500 nm). Sodium acetate (CH_3_COONa) and sodium sulfate (Na_2_SO_4_) were supplied by Duksan Pure Chemicals Co. Ltd., South Korea. Ethyl cellulose and α-terpineol (C_10_H_18_O) were acquired from KANTO Chemical Co., Japan. Fluorine-doped transparent conducting oxide glass (FTO; F-doped SnO_2_ glass; 7 Ω sq^−1^) was supplied by Pilkington, USA. All the above reagents used in this study were of analytical grade and used as received. All solutions were prepared from DI water obtained using a PURE ROUP 30 water purification system.

### Facile synthesis stages for CeO_2_-Graphene nanostructures

**Figure 12:**
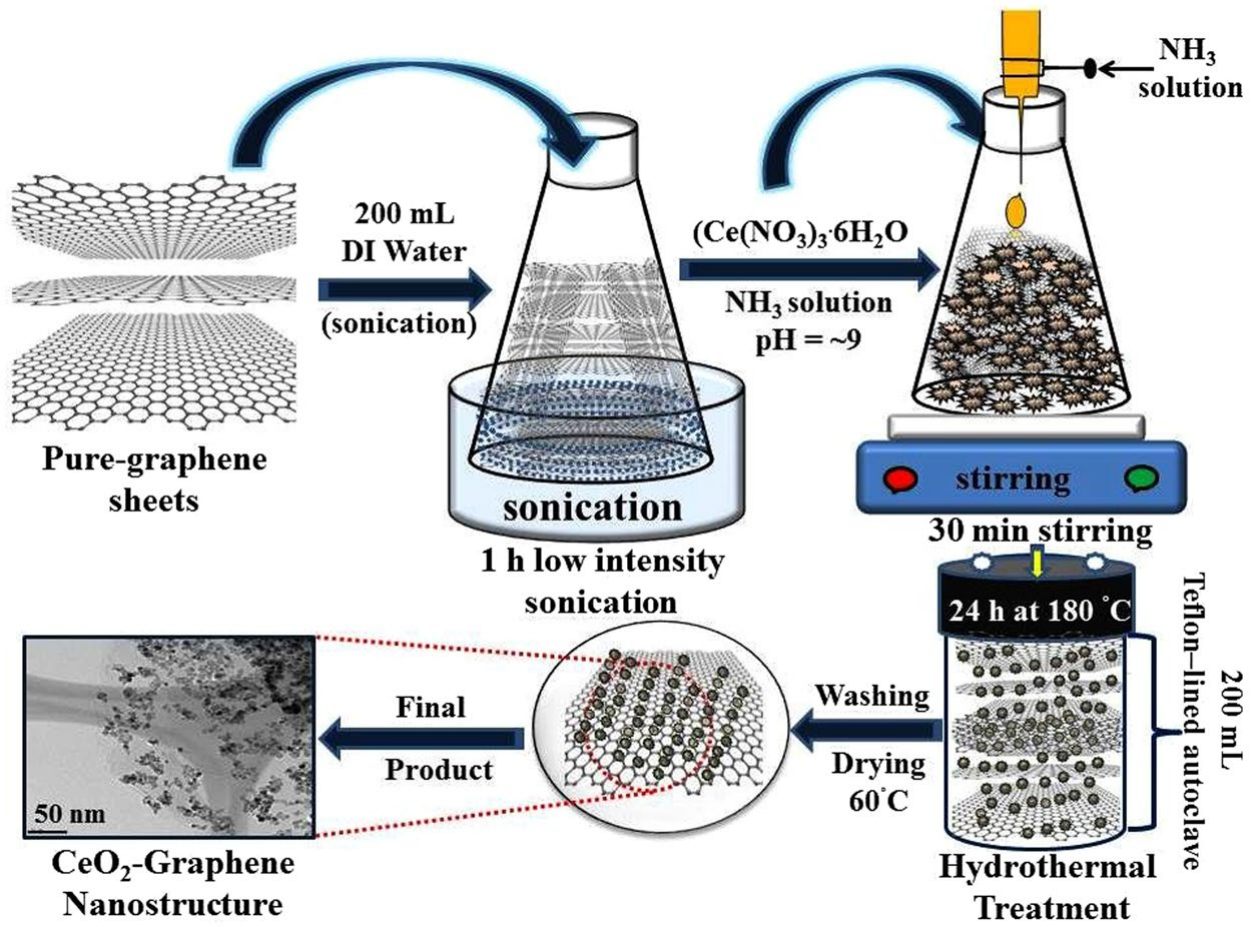
Schematic representation for the synthesis steps of the CeO_2_-Graphene nanostructures.

In general, there are two different procedures to design and control the synthesis of graphene-based nanostructures. In the first procedure, the precursor salt adsorbs on the surface of the graphene matrix and nanoparticles are grown directly by controlling the size and shapes (*in situ* growth), while in the second procedure, the as-synthesized nanostructures are anchored/deposited onto the surface of the graphene matrix (self-assembly approach)^65^ (Fig. 12).

Herein, a simple, low cost and single step (*in situ* growth) hydrothermal method was used for the controlled growth of CeO_2_ nanoparticles on graphene sheets. Scheme 1 presents a schematic diagram of the mechanism for the fabrication of CeO_2_-Graphene nanostructure. The CeO_2_-Graphene nanostructures were synthesized using a simplistic hydrothermal method. Briefly, in the first step, 100 mg of optimal pure-graphene nanopowder was dispersed in 200 mL of de-ionized water and subjected to a low sonication system (Branson 2800, low power sonication instrument) for 1 h. Subsequently, 0.4322 g (Ce(NO_3_)_3_ **·**6H_2_O) of 1 mM aqueous solution was added slowly to the above graphene dispersion. 1 mL of aqueous ammonia solution was added drop wise to the above reaction mixture with constant stirring to maintain the pH value of reaction ~9. The resulting reaction mixture was stirred vigorously for 1 h, poured into a 100 mL of Teflon-lined stainless steel autoclave tube and kept in an electric oven for 24 h at 180 °C to perform the hydrothermal treatment. Once the reaction was accomplished, the autoclave was cooled naturally to room temperature. The product was washed several times with de-ionized water and absolute ethanol, and dried overnight in a vacuum at 60 °C. Finally, a black color powder was obtained. A 3 mM CeO_2_-Graphene nanostructure sample was also synthesized using the same amount of precursor as the above stated procedure.

### Photocatalytic degradation measurements of CR and MB using 1 mM and 3 mM CeO_2_-Graphene nanostructures

The photocatalytic degradation ability of the as-synthesized 1 mM and 3 mM CeO_2_-Graphene nanostructures and pure-graphene samples were assessed for the photocatalytic degradation of CR and MB as a commercially available model pollutant dyes and the rates of degradation were calculated. In the experiment, a 2 mg sample of each photocatalyst was suspended in 25 mL of an aqueous model dye solution (concentration = 5 mg L^−1^) and each solution was sonicated 10 min for homogeneous mixing. The dye solutions were stirred in the dark for 30 min to complete the adsorption and desorption equilibrium of the specific substrate on the CeO_2_-Graphene nanostructure and pure-graphene. Visible light irradiation of the solutions was performed using a 400 W lamp (λ > 500 nm). Three sets of experiments were observed over a 120 min period. The rate of dye degradation was monitored by taking 2 mL of the samples from each set every 30 min, centrifuging, removing the catalyst, and recording the UV–vis spectrum. As a control experiment, pure-graphene was used and considered as the reference photocatalyst. Each experiment was performed in triplicate to ensure the photocatalytic activities of the CeO_2_-Graphene nanostructure.

### Stability and reusability tests for the 1 mM and 3 mM CeO_2_-Graphene nanostructures

Stability and reusability are substantial factors that allow an understanding of the practical efficacy of photocatalysts. The preliminary tests for the stability and the effectiveness were performed by suspending CeO_2_-Graphene nanostructures powder in water and sonicating it for 1 h. The CeO_2_ NPs leaching in the solution, if any, were observed using a UV–vis spectrophotometer, which confirmed the constancy of the CeO_2_-Graphene nanostructure and the possibility of using them as a reusable catalyst (Fig. S1). In another test, a triplicate check of the reusability tests of the CeO_2_-Graphene nanostructures for CR and MB were tested after centrifuging the catalyst from the model dye solution. The recovered catalyst was washed with de-ionized water, dried in an oven at 60 °C, and reused for a second and third run to evaluate its photocatalytic ability with the model dye solution under the same conditions (Fig. S2(a and b)).

### Photocapacitive measurements using CV and EIS of pure-graphene, 1 mM and 3 mM CeO_2_-Graphene nanostructures

The photocapacitive performance of the pure-graphene, 1 mM and 3 mM CeO_2_-Graphene nanostructures were examined by CV and EIS under ambient conditions in the dark and under visible light irradiation. The CV experiment was performed in 50 mL of 0.1 M PBS in the dark and under visible light irradiation at a scan rate of 50 mV s^−1^. EIS was conducted in 50 mL of an aqueous 0.2 M Na_2_SO_4_ solution in the dark and under visible light irradiation at room temperature. The photocapacitive response was examined by CV in the dark and under visible light irradiation at a scan rate of 50 mV s^−1^ over the potential range, 1.0 to −1.0 V. EIS was performed in the dark and later under visible light irradiation (λ > 500 nm) with frequencies ranging from 1 to 10^4^ Hz at 0.0 V vs. Ag/AgCl in potentiostatic mode.

### Standard characterization techniques

The structure of the as-prepared samples and their crystallite size were confirmed by X-ray diffraction (XRD, PANalytical, X’pert PRO-MPD, Netherlands) using Cu Kα radiation (λ = 0.15405 nm). The peaks of the crystalline phases were matched to the standard compounds reported in the JCPDS data file, Raman spectroscopy (Lab Ram HR800 UV Raman microscope; Horiba Jobin-Yvon, France) was performed to confirm the synthesis of the CeO_2_-Graphene nanostructure. The optical properties of the CeO_2_-Graphene nanostructure were examined by UV-VIS-NIR diffuse absorbance/reflectance spectrophotometry (VARIAN, Cary 5000, USA). Electron paramagnetic resonance (EPR) measurements were performed using a Bruker EMX system with a microwave frequency of 9.64 GHz and a microwave power of 1 mW at room temperature. Microstructural analysis was observed by field emission transmission electron microscopy (FE-TEM, Tecnai G2 F20, FEI, USA) operating at an accelerating voltage of 200 kV. Selected-area electron diffraction (SAED) was carried out by TEM. The elemental mapping of the sample containing the phases with different valences was obtained by TEM. Quantitative analysis was performed by energy dispersive spectrometry (EDS). The specimens for TEM and HRTEM measurements were prepared by drop-casting a droplet of ethanol suspension onto a copper grid coated with a thin amorphous carbon film, and dried in air. The BET specific surface area of the samples was measured using a Belsorp II-mini (BEL, Japan Inc.). X-ray photoelectron spectroscopy (XPS, ESCALAB 250 XPS System, Thermo Fisher Scientific U.K.) was conducted using the following X-ray source: monochromatic Al Kα, hν = 1486.6 eV, X-ray binding energy (BE) of 15 kV, 150 W and spot size of 500 μm; take-off angle of 90°; Pass energy of 20 eV; and BE resolution of 0.6 eV. All the BE values were calibrated using the BE of C 1s (284.6 eV). XPS fitting was performed using “AVANTAGE” software with a Shirley subtraction and the shape of the peaks used for deconvolution was Gaussian–Lorentzian. XPS was conducted at the Korea Basic Science Institute (KBSI), South Korea. The photocatalytic and photoelectrochemical experiments were carried out using a 400 W lamp (3 M, USA) with λ > 500 nm and an intensity of 31 mW cm^−2^.

### Photocatalytic degradation tests and photocapacitance measurements

The photocatalytic degradation of the organic pollutants process was monitored by measuring the absorption of the organic model pollutants by UV–vis spectrophotometry (OPTIZEN 2120UV). The photoelectrochemical studies, such as cyclic voltammetry (CV) and electrochemical impedance spectroscopy (EIS), were performed using a potentiostat (Versa STAT 3, Princeton Research, USA) comprised of a standard three-electrode system. Ag/AgCl (3 M KCl), a Pt gauge and FTO glass coated with pure-graphene and CeO_2_-Graphene nanostructures used as the reference, counter, and working photoelectrodes, respectively. EIS was performed in a 0.2 M Na_2_SO_4_ solution; CV was performed in a 0.2 M phosphate buffer solution (pH 7; 0.2% PBS) as the supporting electrolyte at room temperature. The projection area of the photoelectrode was 1 cm^2^.

### Photoelectrode groundwork

The working electrodes were prepared as follows. A 100 mg mass of each sample was mixed thoroughly by adding ethyl cellulose as a binder and α-terpineol as the solvent. The mixture was stirred instantaneously and heated at hot plate magnetic stirrer until a thick paste was obtained. The obtained paste was then coated on carbon paper using the doctor-blade method and kept drying under a 60 W lamp overnight and the electrode was later used as a photoelectrode.

## Electronic supplementary material

Supplementary Information
