# Supplementary material for: Ce3+-ion, Surface Oxygen Vacancy, and Visible Light-induced Photocatalytic Dye Degradation and Photocapacitive Performance of CeO2-Graphene Nanostructures
Source: Sci Rep. 2017 Jul 19;7:5928. doi: 10.1038/s41598-017-06139-6 (PMC5517655; doi:10.1038/s41598-017-06139-6)
Supplement: Supplementary file 1 — Supplementary Information [file 41598_2017_6139_MOESM1_ESM.pdf]

Electronic supplementary information

Ce^{3+}-ion, Surface Oxygen Vacancy, and Visible Light-induced Photocatalytic Dye Degradation and Photocapacitive Performance of CeO_2-Graphene Nanostructures

Mohammad Ehtisham Khan[1], Mohammad Mansoob Khan[2**], and Moo Hwan Cho[1*]

[1]*School of Chemical Engineering, Yeungnam University, Gyeongsan-si, Gyeongbuk 38541, South Korea. Phone: +82-53-810-2517, Fax: +82-53- 810-4631.*
[2]*Chemical Sciences, Faculty of Science, Universiti Brunei Darussalam, Jalan Tungku Link, Gadong, BE1410, Brunei Darussalam.*

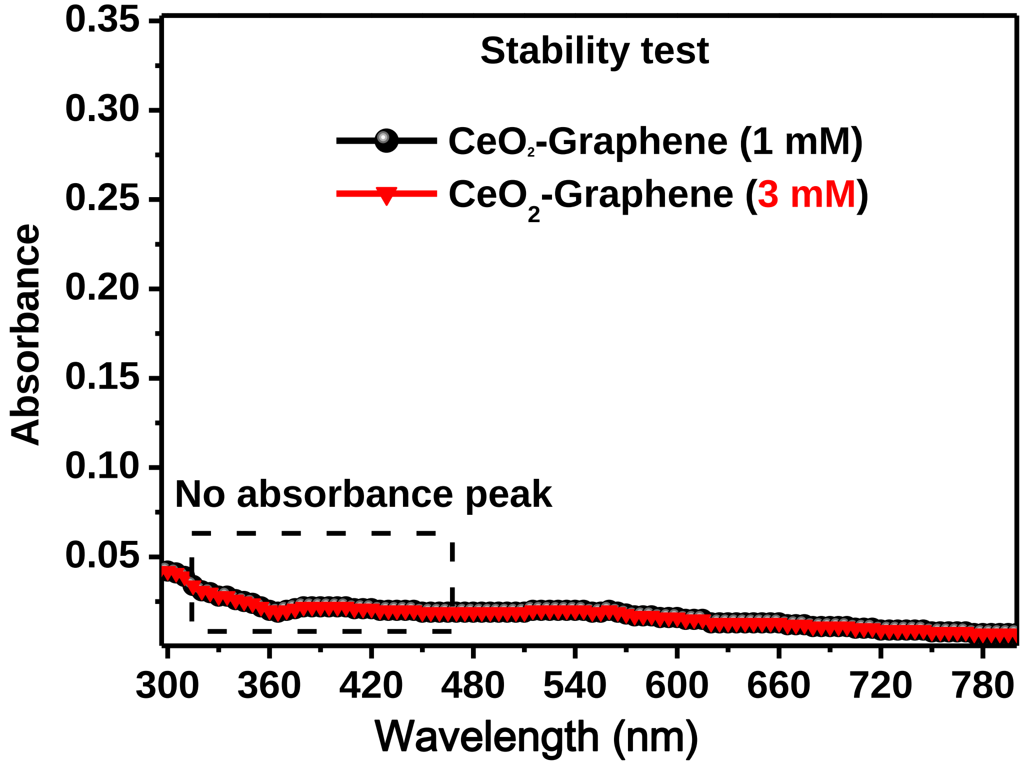

Fig. S1 Stability test of 1 mM and 3 mM CeO_2-Graphene nanostructures.

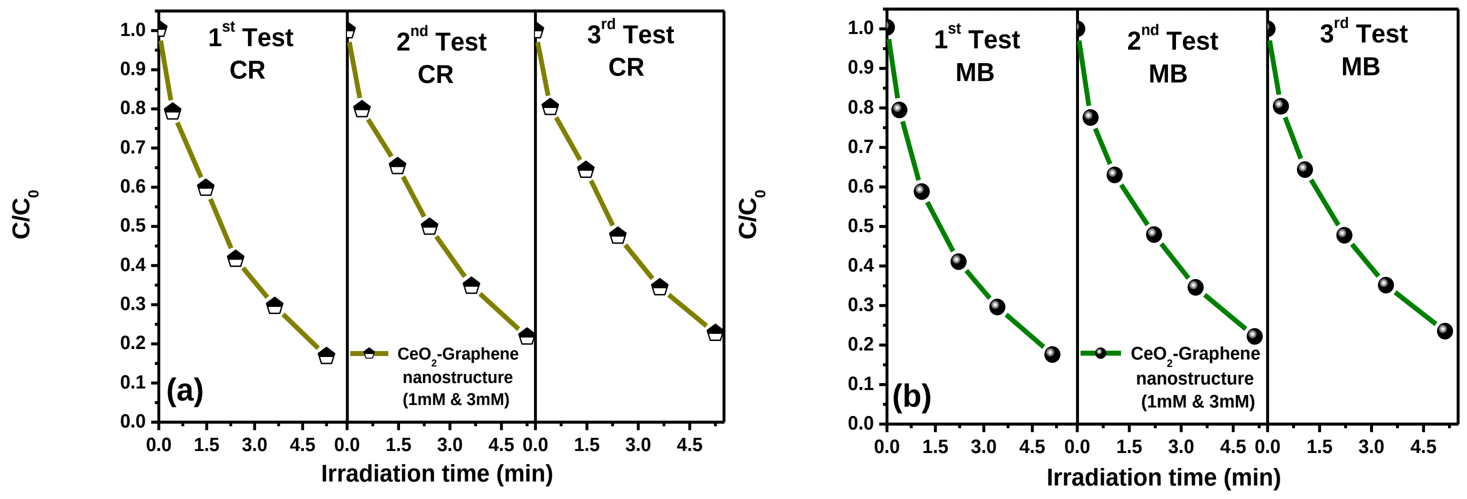

Fig. S2 (a and b) CR and MB reusability test spectra of 1 mM and 3 mM CeO_2-Graphene nanostructures.

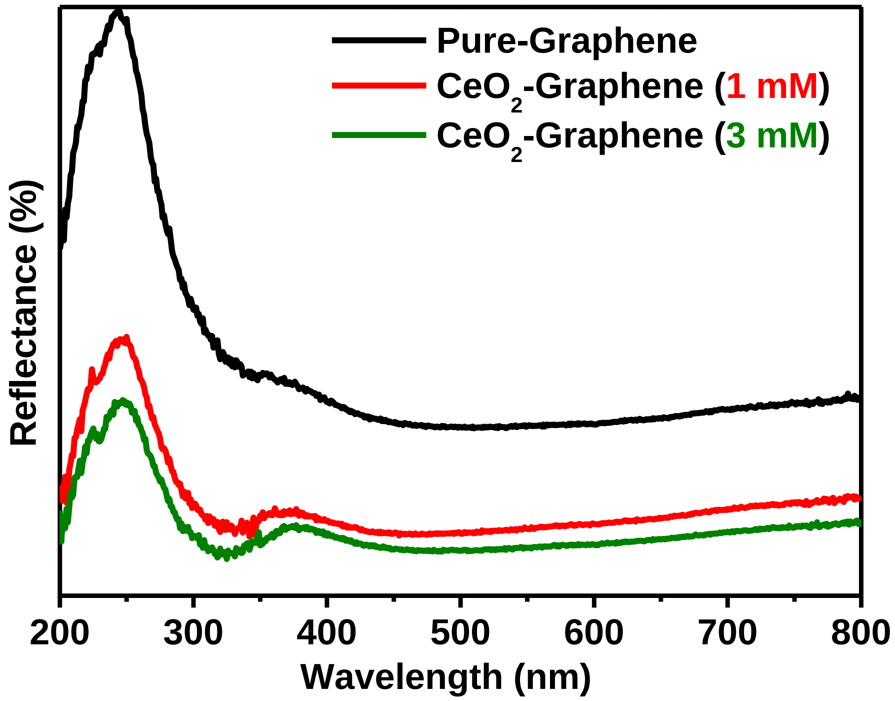

Fig. S3 UV-Vis reflectance spectra of pure-graphene, 1 mM and 3 mM CeO_2-Graphene nanostructures.

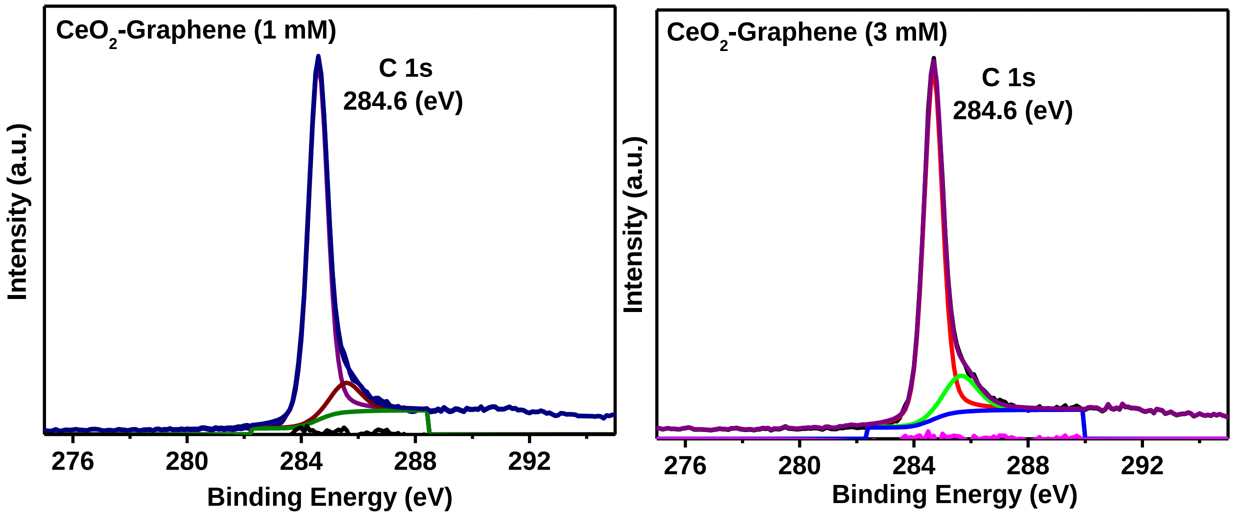

Fig. S4 (a and b) Fitted peak of C 1s corresponds to 1 mM and 3 mM CeO_2-Graphene nanostructure.

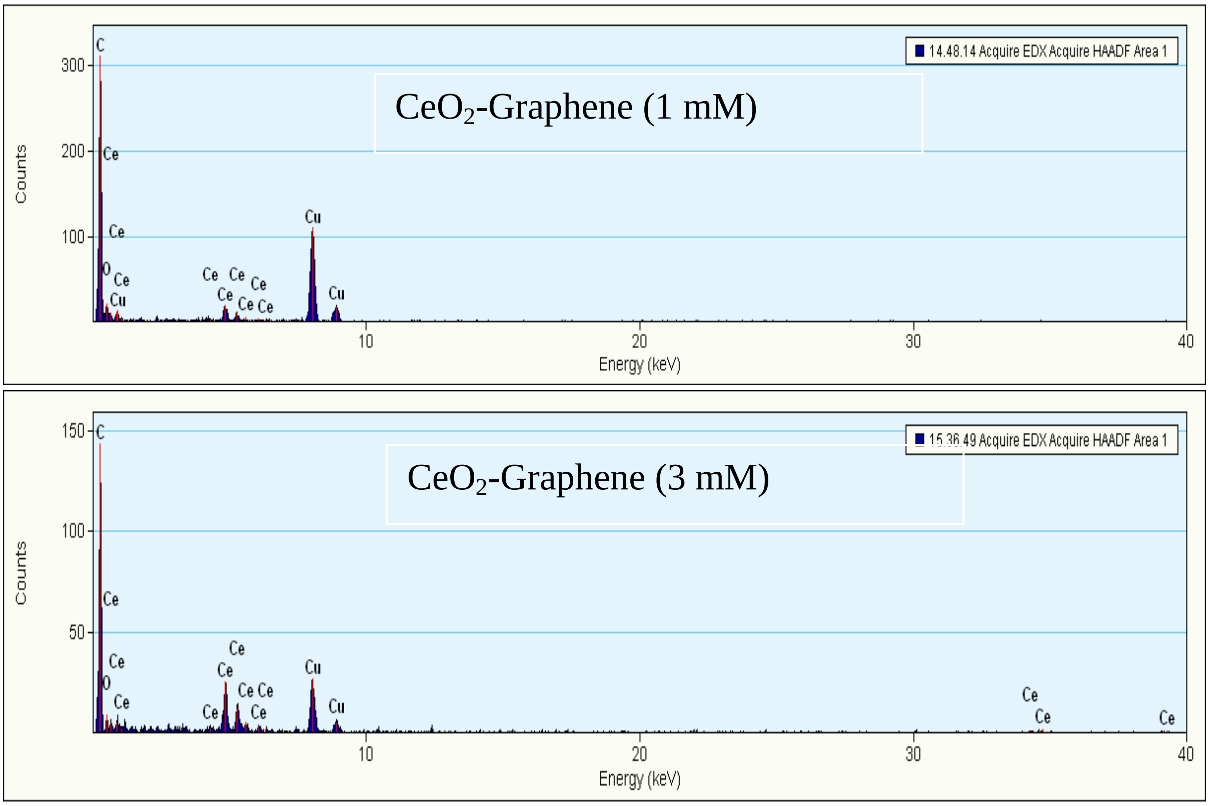

Fig. S5 (a and b) Elemental composition of 1 mM and 3 mM CeO_2-Graphene nanostructure.

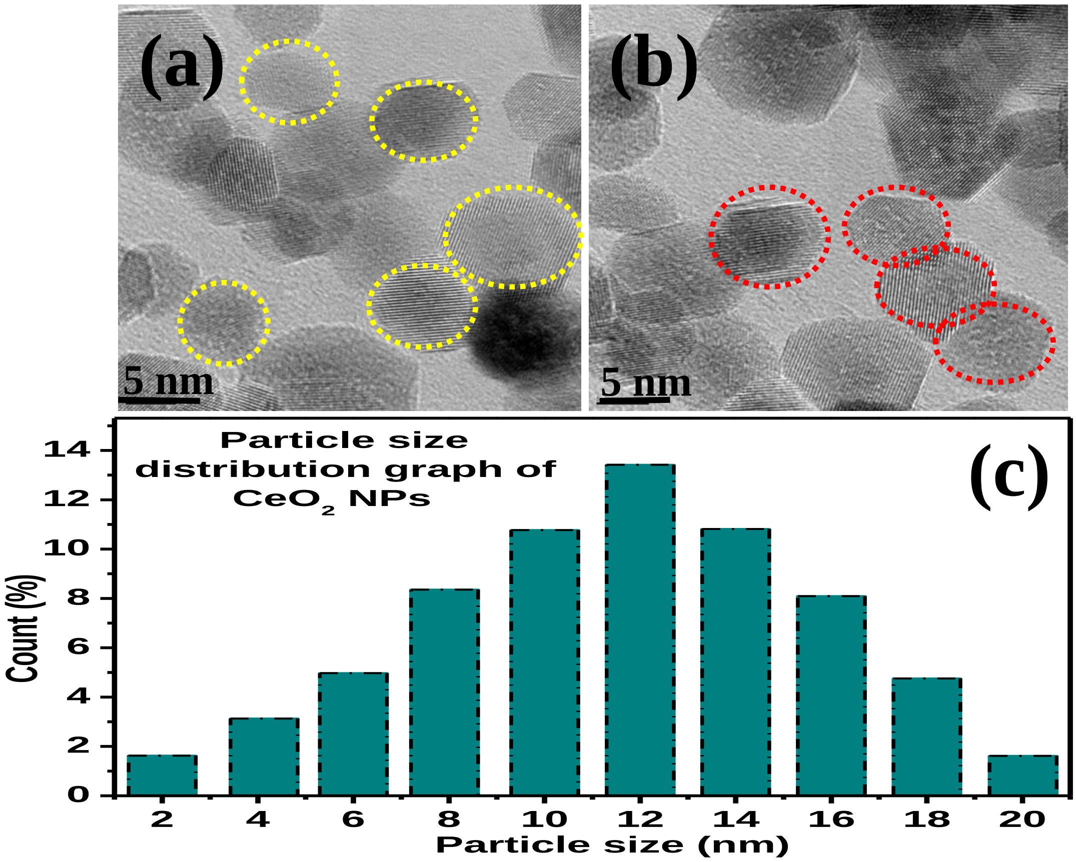

Fig. S6 (a and b) HR-TEM images of 1 mM and 3 mM CeO_2 NPs with high magnification values and visible lattice fringes **(c)** particle size distribution graph of CeO_2 NPs.
